# Tunable N_2_ Fixation Enabled by Ferroelectric Switching in Doped Graphene/In_2_Se_3_ Dual-Atom Catalysts

**DOI:** 10.1021/acsami.4c21092

**Published:** 2025-02-27

**Authors:** Mohammad Amin Akhound, Maryam Soleimani, Mahdi Pourfath

**Affiliations:** † School of Electrical and Computer Engineering, College of Engineering, University of Tehran, Tehran 14395-515, Iran; ‡ CAMD, Department of Physics, Technical University of Denmark, DK - 2800 Kongens Lyngby, Denmark; ¶ Dipartimento di Scienza dei Materiali, Università di Milano − Bicocca, via R. Cozzi 55, 20125 Milano, Italy; § Institute for Microelectronics/E360, TU Wien, A-1040 Vienna, Austria

**Keywords:** nitrogen reduction reaction, dual-atom catalysts, ferroelectric materials, density functional theory, two-dimensional materials, α-In_2_Se_3_, electrocatalysis

## Abstract

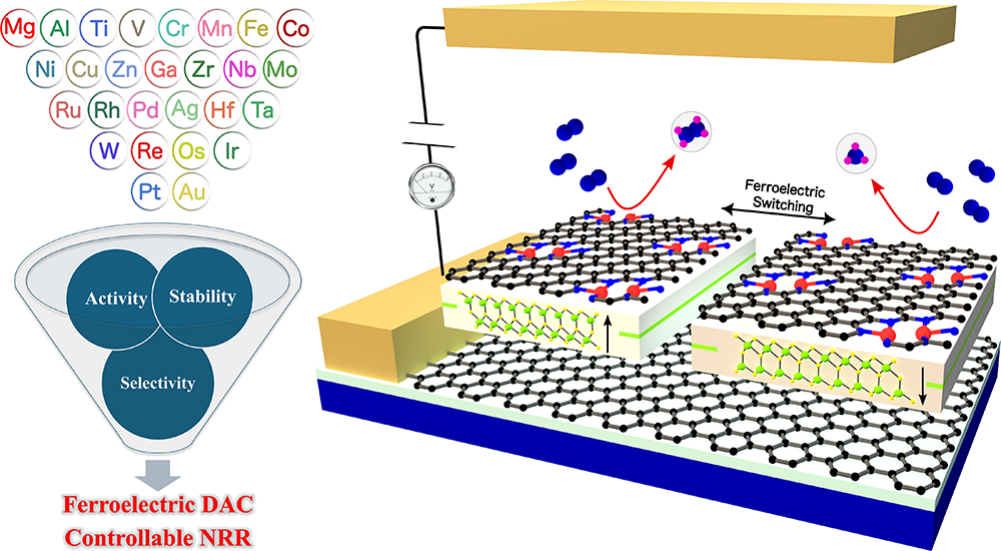

The electrochemical nitrogen reduction reaction (NRR) provides a sustainable alternative to ammonia synthesis. However, the development of catalysts with high activity and selectivity under ambient conditions remains a significant challenge. In this work, we propose a class of dual-atom catalysts (DACs), consisting of two metal atoms embedded in nitrogen-doped porous graphene (M_2_NPG) supported on a ferroelectric α-In_2_Se_3_ monolayer. Using density functional theory (DFT) calculations, we explore the effect of ferroelectric polarization switching on the structural stability, catalytic performance, and reaction mechanisms of these DACs. By computationally screening 27 metal atoms as active sites, we identify four promising candidates (V, Co, Ru, and Ta) with V_2_NPG@In_2_Se_3_ standing out due to its exceptional properties. The precise control of NRR pathways, along with tunable limiting potentials and selective product formation, can be achieved through the polarization switching of the α-In_2_Se_3_ monolayer. The combination of low limiting potential, abundant active sites, tunable catalytic behavior, and high selectivity against the hydrogen evolution reaction (HER) highlights the potential of V_2_NPG@In_2_Se_3_ as a promising alternative to traditional single-atom catalysts. This work demonstrates a versatile strategy for integrating DACs with ferroelectric materials, offering valuable insights into designing next-generation catalysts for NRR and beyond.

## 1 Introduction

The growing demand for sustainable energy solutions, driven by the rapid depletion of fossil fuel reserves, has accelerated the pursuit of green energy technologies. Among these, the electrochemical conversion of nitrogen (N_2_) to ammonia (NH_3_) is a sustainable alternative to the energy-intensive Haber-Bosch process.^1−5^ However, efficient catalysts for the nitrogen reduction reaction (NRR) remain a challenge, especially for practical applications. The reaction kinetics are notably slow, and the process is highly competitive with the hydrogen evolution reaction (HER) in aqueous environments.^6,7^ This competition severely limits the Faradaic efficiency (FE) of various catalysts, including metals,^8,9^ metal oxides,^10,11^ and transition metal chalcogenides,^12−14^ resulting in FE values often well below optimal levels.

Single-atom catalysts (SACs), which consist of isolated metal atoms anchored on two-dimensional (2D) materials such as graphene or metal oxides, have shown great promise in addressing these challenges due to their high atom efficiency and precise control over catalytic processes.^15,16^ These catalysts enhance the NRR by utilizing isolated single-atom active sites, where the electronic properties of the metal atoms are carefully tuned through their coordination environment. This enables effective nitrogen activation and supports ammonia synthesis.^17^ Despite their advantages, SACs face challenges related to atom stability, as single metal atoms tend to aggregate under reactive conditions, diminishing their catalytic effectiveness. The performance of SACs is significantly influenced by the choice of the support material, which complicates their synthesis and limits their practical applications. Moreover, accurately characterizing single atoms remains a major challenge, further hindering their development for real-world applications.^18,19^

However, dual-atom catalysts (DACs) offer a promising solution by providing multiple active sites that can facilitate different reaction steps independently, optimizing each atom’s role. This separation helps to overcome scaling relationships that limit the activity of SACs, which struggle to balance the binding energies of the adsorbates. Therefore, DACs achieve improved activity and stability compared to SACs.^20^ Recent studies have revealed that DACs can achieve significantly higher ammonia yield rates and FEs compared to SACs.^21−25^

Despite recent progress, there is still significant potential for developing novel catalysts with improved controllability, selectivity, and activity for large-scale industrial applications. 2D ferroelectric materials, such as α-In_2_Se_3_, have exhibited promising performance by switching polarization and modulating catalytic activity by adjusting electronic structure.^26,27^ The unique characteristic of ferroelectric materials enables precise control over catalytic processes and enhances activity and selectivity compared with those of traditional catalysts. One study demonstrated that doping transition metal atoms onto ferroelectric α-In_2_Se_3_ monolayers (TM@In_2_Se_3_) can improve CO_2_ reduction reactions by altering reaction pathways and final products through polarization switching.^28^ The same ferroelectric switching effect has been observed for the electrocatalytic oxidation of CO on α-In_2_Se_3_ with selenium vacancy defects, doped with single metal atoms.^29^ The study of ferroelectric heterostructures, such as Mo-BN@In_2_Se_3_ and WSe_2_@In_2_Se_3_, exhibits either semiconducting or metallic properties based on polarization direction due to a built-in electric field and electron transfer, optimizing both photocatalytic and electrocatalytic reactions.^30^ Furthermore, recent research indicates that heterostructures of graphene doped with single metal atoms on a ferroelectric layer, such as CoN_3_ on α-In_2_Se_3_, show promising improvements in HERs due to polarization-induced electron redistribution and band state shifts, leading to better catalytic efficiency and stability.^31^ These developments highlight the significant potential of 2D ferroelectric materials for the creation of next-generation controllable electrocatalysts.

While previous research on ferroelectric materials has focused solely on SACs, our study introduces a novel class of 2D heterostructures for NRR that integrate dual metal atoms with controllable ferroelectric properties. To achieve this, N-doped porous graphene containing metal dimers (M_2_NPG) with excellent synthesizability^32−35^ and superior NRR activity^36,37^ is placed on top of the ferroelectric α-In_2_Se_3_ monolayer.^38,39^ Through a systematic investigation of 27 metal atoms, we identify four metal atoms as highly stable active sites with promising catalytic performance. Among these, V_2_NPG@In_2_Se_3_ demonstrates the ability to control reaction barriers, pathways, and final products of the NRR via polarization switching. The significance of this work lies in the achievement of a highly active, selective, and controllable catalyst that not only leverages the unique features of ferroelectric materials but also harnesses the advantages of DACs, including numerous active sites.

## 2 Methods and Computational Details

All calculations were performed using the spin-polarized density functional theory (DFT) methods, implemented through the Vienna Ab initio Simulation Package (VASP) software.^40,41^ PBE-GGA spin-polarized approximation treats the exchange-correlation interactions, while the frozen-core projector augmented wave (PAW) approximation describes the interaction between ion and electron.^42−44^ The Hubbard *U* parameter is not included in this study; however, a detailed analysis of the effect of the *U* parameter on the results can be found in the Supporting Information Section 1.^45−51^ The long-range van der Waals (vdW) interactions between layers are described by the semiempirical DFT-D3 method in Grimme’s scheme.^52^ The NPG@α-In_2_Se_3_ heterostructure is created by vertically stacking a 5 × 5 × 1 NPG supercell^53^ on a 3 × 3 × 1 α-In_2_Se_3_ monolayer. The chosen supercell size for the NPG layer is well-established in the literature for DACs and ensures sufficient separation between periodic dual-atom sites, thereby minimizing artifacts arising from periodic boundary conditions.^54,55^ To prepare the NPG layer, four carbon atoms were removed from the graphene center to create vacancies, which can accommodate the metal dimer. The six unsaturated carbon atoms surrounding the vacancies were replaced with nitrogen atoms to form the nitrogen-doped porous graphene structure.^34,35,56^ The M_2_NPG@α-In_2_Se_3_ heterostructure was then constructed by incorporating two metal atoms into the vacancy sites.

The lattice parameter of the NPG@α-In_2_Se_3_ heterostructure is 12.3 Å, which aligns well with previous studies.^31,57,58^ The lattice mismatch in this structural model is 0.15%, which minimally impacts the computed total energies as well as the structural and electronic properties. A vacuum spacing of over 20 Å is introduced perpendicular to the surface to prevent interactions between periodic layers. The dipole correction is also applied to all asymmetric structures.^59^ An energy cutoff of 500 eV is adopted for the plane-wave basis. In structural optimizations, the Brillouin zone is sampled with 5 × 5 × 1 *k*-points using the γ-centered *k*-mesh, while the denser *k*-points of 10 × 10 × 1 are used for electronic properties computations. The energy and force convergence thresholds for the self-consistent field (SCF) iteration are set to 10^–5^ eV and 0.01 eV/Å, respectively. The climbing image nudged elastic band (NEB) method^60^ is employed to determine the transition state during the reactions.

Thermal stability was assessed through ab initio molecular dynamics (AIMD) simulations at room temperature (298.15 K), with the Nose-Hoover thermostat ensuring consistent temperature control.^61^ The simulation ran for 5 ps with a time step of 1 fs, providing insights into the stability of the structure over time. In addition, the free energy diagram of the NRR is obtained using the computational hydrogen electrode (CHE) model proposed by No̷rskov et al.^62^ The free energy difference for each elemental reaction step is calculated as^63,64^
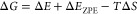
1where Δ*E* represents the electronic energy difference obtained directly from DFT calculations, Δ*E*_ZPE_ denotes the change in zero-point energies, *T* is the temperature (set at 298.15 K), and Δ*S* is the entropy change. The effect of solvation on the free energy diagrams of the final candidates is further investigated in the Supporting Information Section 2.^65^ Furthermore, grand canonical DFT calculations are performed, and the results are compared with constant-charge DFT calculations, which are further discussed in the Supporting Information Section 3.^66−68^ The charge density difference (CDD) is calculated to evaluate the charge transfer between the surface and N_2_ molecule using the following equation^69^:
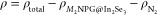
2where ρ_total_, ρ_M_2_NPG@In_2_Se_3__, ρ_N_2__ are the charge of N_2_ molecule adsorbed on the M_2_NPG@In_2_Se_3_, free M_2_NPG@In_2_Se_3_ and isolated N_2_ molecule, respectively.

## 3 Results and Discussion

### 3.1 Structural Models of Ferroelectric DACs

Different from ferroelectric SACs,^28,29,31^ this study introduces a novel DAC where a 2D layer of M_2_NPG is placed on top of a ferroelectric α-In_2_Se_3_ monolayer. Constructing a heterostructure rather than doping metal atoms directly on α-In_2_Se_3_ has several advantages. First, DACs cannot be achieved through direct doping; single-atom doping is typically limited to isolated metal atoms rather than pairs, which limits the catalytic versatility. Moreover, doping metal atoms into α-In_2_Se_3_ would likely face synthetic challenges, as creating homogeneously dispersed dopant sites on a layered ferroelectric substrate is complex and can lead to structural distortions or clustering, reducing catalytic efficiency and consistency.

In contrast, constructing a heterostructure with M_2_NPG provides a more controllable platform for positioning dual metal atoms, facilitating easier synthesis and ensuring consistent active sites. Additionally, heterostructures inherently possess enhanced interfacial effects, allowing for fine-tuning of the catalytic properties through the interaction between NPG and the underlying polarized α-In_2_Se_3_. This layered approach leverages the intrinsic polarization of α-In_2_Se_3_ to modulate the electronic properties in M_2_NPG, creating a flexible and effective DAC design for the NRR and overcoming limitations commonly faced in directly doped systems.^70^

The choice of α-In_2_Se_3_ as the bottom layer is based on its robust stability at room temperature and its experimentally demonstrated switchable polarization, providing a unique platform for designing controllable catalysts.^28,30,39,71^ Furthermore, this material’s versatility has also been highlighted in studies on tunable electronic and magnetoelectric devices, showcasing its potential for multifunctional applications.^58,72,73^ This material consists of five covalently bonded monatomic layers arranged as Se–In–Se–In–Se, forming a triangular lattice.^38^ The middle Se atoms are asymmetrically bonded to four neighboring In atoms, creating a noncentrosymmetric structure essential for ferroelectricity. The calculated lattice constant of monolayer α-In_2_Se_3_ is *a* = 4.058 Å, consistent with previous studies.^72−74^ According to the electrostatic surface potential (ESP) of the α-In_2_Se_3_ monolayer, shown in Figure S1a, there is a significant asymmetry between the two surfaces of this material, with a potential difference of 1.23 eV. This disparity indicates the presence of an intrinsic electric field across the monolayer, which is proof of spontaneous polarization in ferroelectric materials. The electric dipole moment is 0.095 eÅ per α-In_2_Se_3_ unit cell. The built-in electric field is highly advantageous for catalytic applications, as it can influence the adsorption and activation of reactant molecules by modifying the electronic environment at the catalyst surface.

Despite its advantageous ferroelectric properties, the sizable band gap of 1.46 eV in α-In_2_Se_3_ hinders its direct application in NRR.^75^Figure S2 illustrates that N_2_ adsorption on both the P↑ and P↓ surfaces of the α-In_2_Se_3_ monolayer is weak, with minimal adsorption energy and a bond length of 1.113 Å, similar to the gas phase of the N_2_ molecule. This physical adsorption behavior suggests that pristine α-In_2_Se_3_ alone is ineffective for activating N_2_, as it lacks the ability to engage in the necessary electron transfer to weaken the N≡N bond. Typically, transition-metal atoms are introduced as catalytic sites due to their partially filled *d* orbitals, which can accept the electron density from N_2_ and back-donate to its antibonding orbitals, effectively facilitating bond weakening. Although main-group metal elements are rarely explored as catalytic active sites, modulating the s/p-band filling of these metals can also lead to enhanced catalytic activity.^76^ Therefore, we introduced a dual-atom-metal-doped nitrogen porous graphene (M_2_NPG) layer atop α-In_2_Se_3_ to leverage these mechanisms. M_2_NPG offers a high density of active sites with tunable metal *d* orbitals, enabling strong hybridization with nitrogen molecular orbitals and promoting electron transfer to destabilize the N≡N bond. Furthermore, this configuration has been successfully synthesized with various metals and is recognized for its robust catalytic performance in NRR.^32−37^

**Figure 1:**
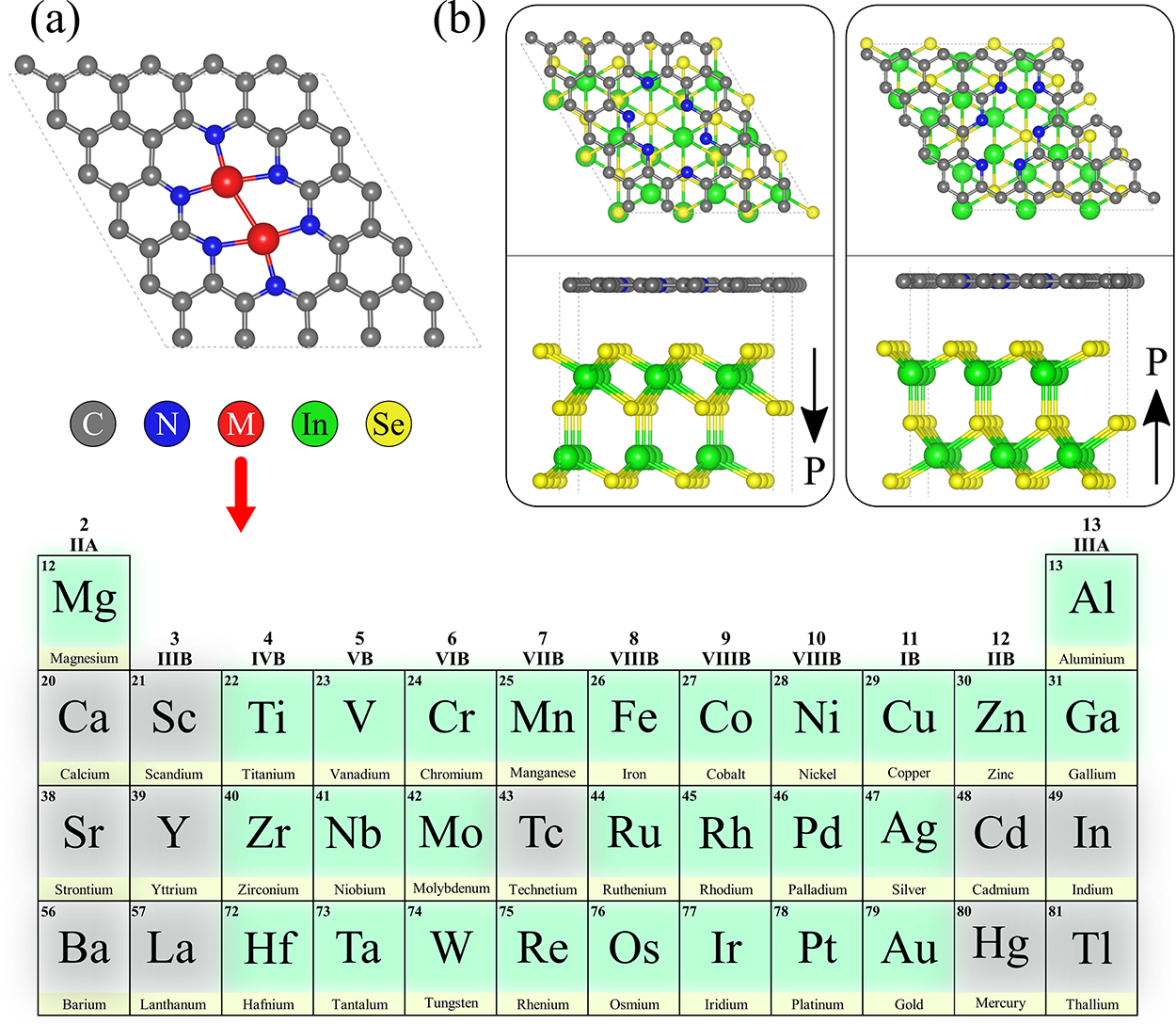
(a) Top view of the 2D M_2_NPG monolayer, highlighting the various metal atoms considered in this study. (b) Top and side views of the optimized NPG@P↓In_2_Se_3_ and NPG@P↑In_2_Se_3_ heterostructures, with black arrows indicating the polarization direction of the α-In_2_Se_3_ monolayer.

Based on the distinct polarization directions of the α-In_2_Se_3_ monolayer surfaces, we identified two heterostructures with ferroelectric polarization oriented either upward (P↑) or downward (P↓), as shown in Figure 1b. The lattice parameters of the α-In_2_Se_3_ monolayer and the NPG layer were carefully selected to ensure minimal strain, maintaining a near-perfect match with only 0.48% of strain applied equally to both layers. This careful selection preserves the structural integrity of the heterostructure and avoids distortions that could impact the electronic and catalytic properties. To further refine the structural model, the interlayer distance between the NPG layer and the α-In_2_Se_3_ monolayer was systematically varied (2.5 3.0, and 3.5 Å) during the initial setup. After structural optimization, the interlayer distances in the NPG@P↓In_2_Se_3_ and NPG@P↑In_2_Se_3_ configurations converged to 3.45 and 3.44 Å, respectively, in agreement with previously reported values.^57,58^ The ESP of these heterostructures, shown in Figure S1b,c, indicates that the direction of polarization influences the electric field within the NPG layer. The magnitudes of the electric dipoles are 1.05 and 0.47 eÅ for the two oppositely polarized systems, respectively. Consequently, this effect alters the electronic states at the interface, allowing for tunable electronic properties that respond to polarization switching.

The projected density of states (PDOS) for the NPG layer on the P↑ and P↓ surfaces of α-In_2_Se_3_ is shown in Figure S3, alongside the PDOS of pristine α-In_2_Se_3_ for reference. Pristine α-In_2_Se_3_ has a wide indirect band gap, measured at 1.46 eV with HSE06 and 0.78 eV with GGA-PBE, showing a clear distinction between In and Se states near the Fermi level, which highlights its semiconducting nature. Interfacing α-In_2_Se_3_ with NPG modifies its electronic structure, inducing metallic properties. This metallic behavior improves the carrier mobility, making it highly beneficial for electrocatalytic reactions. In the P↓ configuration, significant hybridization between the carbon atoms in NPG and the In and Se atoms near the Fermi level is observed. This hybridization, which enhances charge transfer at the interface, suggests that P↓ polarization promotes a more conductive environment in the NPG layer. In the P↑ configuration, however, the PDOS reveals reduced hybridization and fewer states near the Fermi level, implying weaker interaction and lower charge transfer compared to the P↓ case.

To enable efficient catalysis, we incorporated two metal atoms into the vacancy sites within the NPG layer, creating a series of homonuclear DACs, as illustrated in Figure 1a. In principle, all 3d, 4d, and 5d transition metals, as well as main group metals, can serve as metal centers in M_2_NPG. However, due to their toxic or radioactive properties, certain metals were excluded from consideration in this study. We focused on 27 metal atoms, including 24 transition metals (excluding Sc, Y, La, Tc, Cd, and Hg) and 3 main group metals (Mg, Al, and Ga) as central atoms in the 2D M_2_NPG layer. To eliminate potential artifacts arising from stacking order variations, the selected vacancy sites and metal incorporation strategy were designed to ensure symmetry. The vacancy sites were large enough and symmetrically aligned above the α-In_2_Se_3_ monolayer to create a uniform interface. This symmetry ensures that all possible atomic arrangements in the underlying layer are equally accessible to the metal atoms in the NPG layer (top view of Figure 1b). As a result, any rotation or shift of the metal atoms or vacancy sites does not alter the electronic interactions or the catalytic properties. In the following sections, we outline the screening methods used to identify stable ferroelectric DACs and investigate their potential catalytic activity for the NRR.

### 3.2 Stability Screening

Using the abovementioned criteria, 54 heterostructures of M_2_NPG@P↓In_2_Se_3_ and M_2_NPG@P↑In_2_Se_3_ were generated and optimized, as illustrated in Figures S4 and S5, respectively. Table S1 summarizes the structural parameters of these heterostructures. The thermodynamic and electrochemical stabilities of these structures were subsequently evaluated by calculating the formation energy *E*_f_, binding energy *E*_b_, and dissolution potential *U*_diss_, defined as follows^77^^,^^78^:
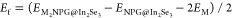
3
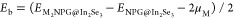
4
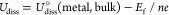
5

Here, *E*_M_ and μ_M_ represent the total energies of a single metal atom from its most stable bulk phase and an isolated metal atom, respectively. *E*_M_2_NPG@In_2_Se_3__ and *E*_NPG@In_2_Se_3__ denote the total energies of the substrate with and without metal atoms.  is the standard dissolution potential of the bulk metal, and *n* is the number of electrons involved in dissolution. According to these definitions, systems with negative *E*_f_ and *E*_b_ are considered thermodynamically stable, indicating that metal atoms prefer to disperse into the vacancy defects of the graphene layer rather than forming metal clusters.^78^ Furthermore, for a structure to be electrochemically stable, the *U*_diss_ vs SHE should be positive.^22^ The exact values of *E*_f_, *E*_b_, and *U*_diss_ can be found in Table S2.

**Figure 2:**
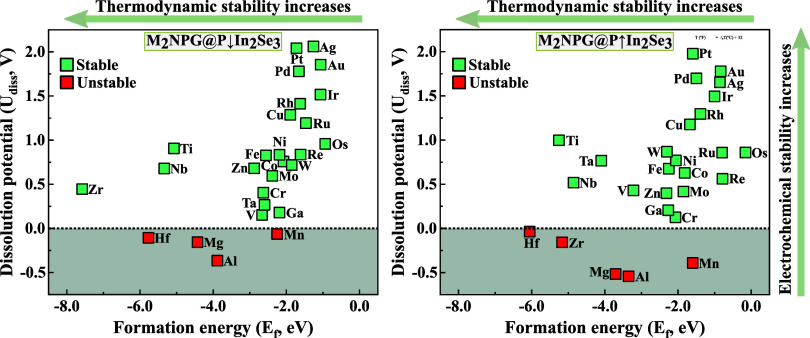
Computed formation energies and dissolution potentials of metal atoms in the M_2_NPG@P↓In_2_Se_3_ and M_2_NPG@P↑In_2_Se_3_ heterostructures.

As shown in Figure 2, all of the investigated metal dimers are thermodynamically stable within the graphene layer, as indicated by their calculated *E*_f_ values well below zero. However, five metal atoms exhibit negative *U*_diss_ values in at least one of the P↑ or P↓ configurations. Since the aim is to control the catalytic reaction by switching polarization directions, it is crucial that the structures remain stable in both orientations. Consequently, ten structures containing Mn, Hf, Zr, Mg, and Al metals embedded in the graphene layer are excluded due to their electrochemical instability in an acidic environment.

### 3.3 N_2_ Adsorption

**Figure 3:**
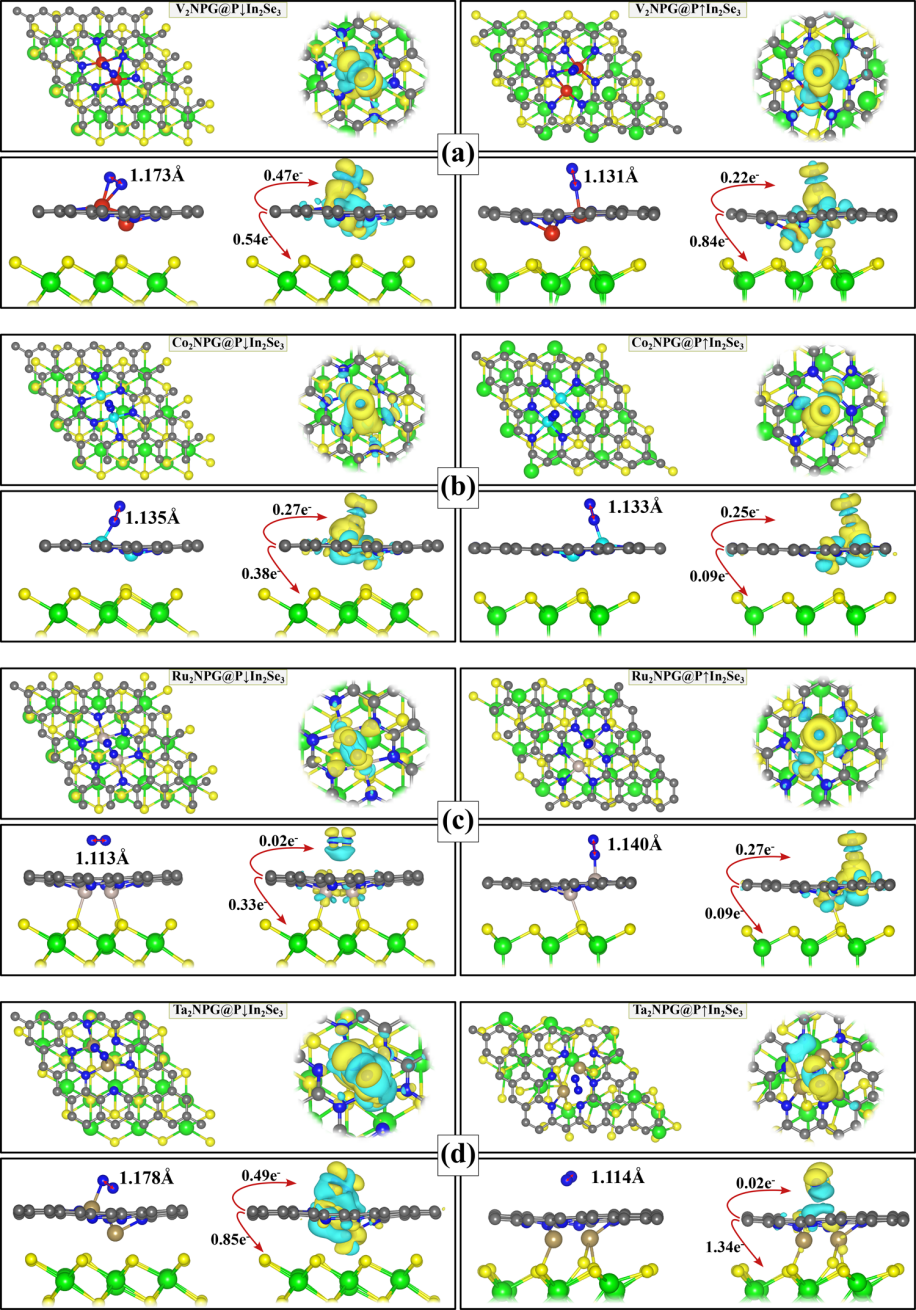
Top and side views of the optimized adsorption configurations and charge density differences of N_2_ molecule on M_2_NPG@P↓In_2_Se_3_ and M_2_NPG@P↑In_2_Se_3_ heterostructures with (a) V_2_, (b) Co_2_, (c) Ru_2_, and (d) Ta_2_ as active sites. The charge depletion and accumulation were depicted by cyan and yellow, respectively. To enhance visualization, different isosurface values are used for the P↓ and P↑ structures, which are (a) 0.0015 and 0.0008 e/Å^3^, (b) 0.0013 and 0.0013 e/Å^3^, (c) 0.0001 and 0.0014 e/Å^3^, and (d) 0.0011 and 0.0001 e/Å^3^, respectively.

The first step in the electrochemical conversion of N_2_ to NH_3_ is the adsorption and activation of the N_2_ molecule. Generally, all stable heterostructures can serve as suitable substrates for the activation of N_2_. To explore the adsorption behavior of N_2_ on the remaining stable heterostructures, three distinct configurations were considered: bridge adsorption, adsorption on top of the first metal atom, and adsorption on top of the second metal atom. The configuration with the lowest adsorption energy was selected as the most favorable site for further analysis. The adsorption energies of N_2_ on 22 metal atoms, for both the P↓ and P↑ surfaces, are provided in Table S3. As shown in this table, 18 metals exhibited either desorption or physical adsorption with adsorption energies greater than −0.25 eV for both the P↓ and P↑ orientations. However, four metal atoms (V, Co, Ru, and Ta) adsorbed N_2_ chemically with notable adsorption energies in at least one orientation. Interestingly, these structures demonstrated distinct adsorption behaviors that were controlled by the ferroelectric polarization of the α-In_2_Se_3_ monolayer. The detailed adsorption properties of the N_2_ molecule on these heterostructures are summarized in Figure 3 and Table 1.

**Table 1:** Adsorption Energies () and Adsorption Configurations () of the N_2_ Molecule on M_2_NPG@In_2_Se_3_ Heterostructures^a^

catalyst	(eV)		(eV)	Δ*G*^H^*^^ (eV)
V_2_NPG@P↓In_2_Se_3_	–0.69	Side-on	–0.10	0.16
V_2_NPG@P↑In_2_Se_3_	–0.51	End-on	–0.03	0.26
Co_2_NPG@P↓In_2_Se_3_	–0.57	End-on	–0.20	0.17
Co_2_NPG@P↑In_2_Se_3_	–0.52	End-on	–0.53	–0.21
Ru_2_NPG@P↓In_2_Se_3_	–0.18	Physisorption	–0.26	0.02
Ru_2_NPG@P↑In_2_Se_3_	–0.61	End-on	–0.37	–0.12
Ta_2_NPG@P↓In_2_Se_3_	–0.99	Side-on	–1.12	–0.88
Ta_2_NPG@P↑In_2_Se_3_	–0.17	Physisorption	–0.14	0.16

a Hydrogen adsorption energies () and Gibbs free energies (Δ*G*^H^*^^) are also included for selectivity comparison.

The activation of N_2_ on M_2_NPG@In_2_Se_3_ surfaces is crucial for the NRR, facilitating electron transfer that weakens the N≡N bond and promotes robust adsorption. This interaction involves both σ and π bonds: the unoccupied *d* orbitals of the metal dimers accept electrons from the σ and π orbitals of N_2_, forming bonding states that stabilize the adsorbed N_2_ molecule. Simultaneously, the occupied *d* orbitals of the metal back-donate electrons into N_2_’s antibonding π* orbitals, weakening the triple bond by populating these antibonding states. Charge density analysis reveals significant electron transfer to the adsorbed N_2_ molecule for V_2_NPG@P↓In_2_Se_3_ and Ta_2_NPG@P↓In_2_Se_3_, with values of 0.47 and 0.49 e charge transfer, respectively, indicating strong side-on N_2_ adsorption configurations.

Polarization switching in M_2_NPG@In_2_Se_3_ induces significant changes in the PDOS of metal *d* orbitals, leading to noticeable shifts in the d-band center (ϵ_d_), as shown in Figure S6. According to the d-band center model, the position of ϵ_d_ relative to the Fermi level governs the interaction strength between metal surfaces and adsorbates, influencing both the adsorption energy and catalytic activity. A higher ϵ_d_ generally correlates with stronger adsorption, as the antibonding states shift higher in energy, reducing their occupation and thus increasing the binding strength. Conversely, a lower ϵ_d_ weakens adsorption by filling antibonding states, leading to a reduced interaction with the adsorbate.

**Figure 4:**
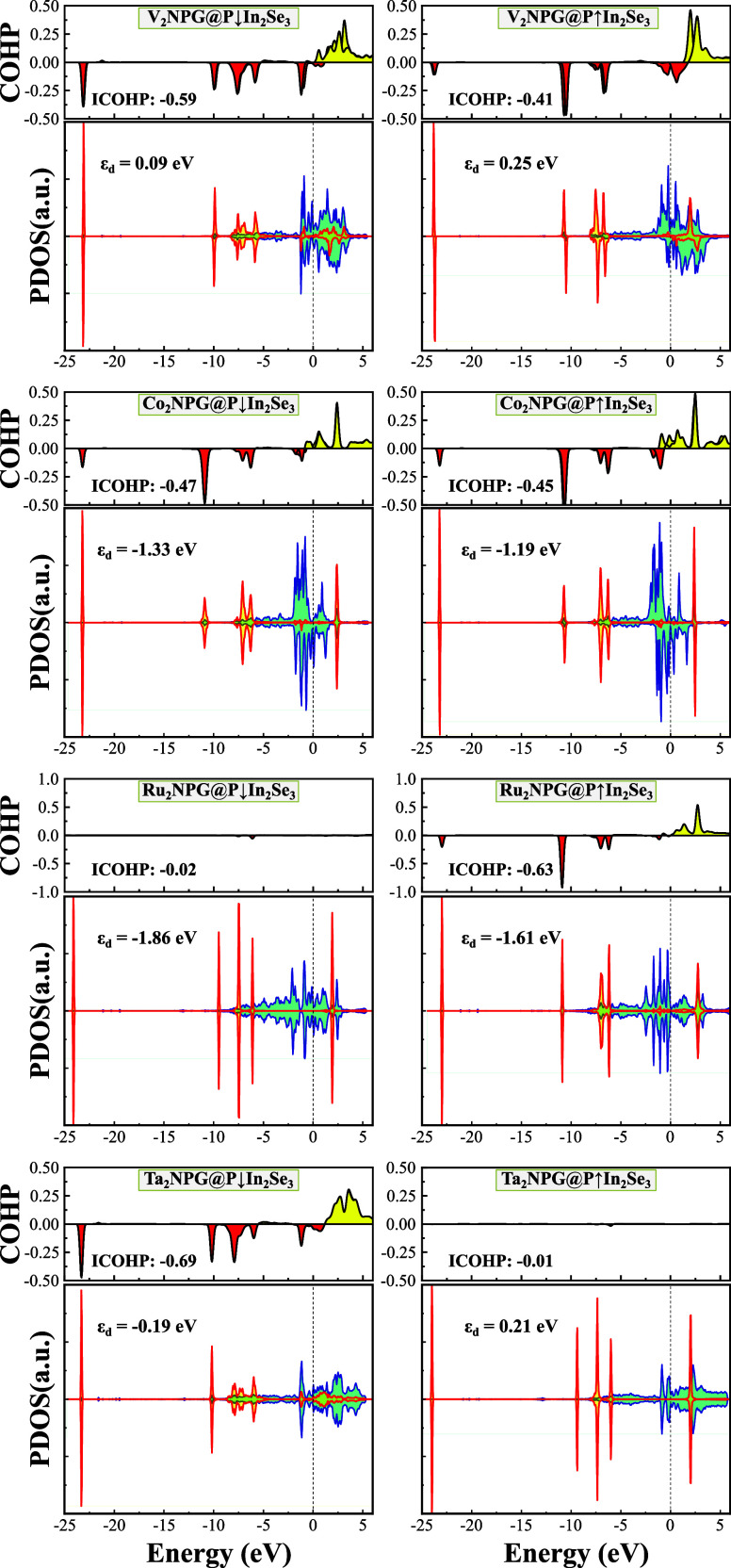
PDOS of N_2_ molecule adsorbed on M_2_NPG@In_2_Se_3_ (M = V, Co, Ru, and Ta) systems with the P↑ and P↓ polarization directions of α-In_2_Se_3_. The blue and red lines correspond to the *d* orbitals of the metal atoms and the *p* orbitals of the N_2_ molecule, respectively. The ϵ_d_ values denote the d-band center of metal atoms relative to the Fermi level, averaged over both the spin-up and spin-down states. Additionally, the COHP of N_2_ on each structure are calculated, with bonding and antibonding states represented by red and yellow, respectively.

The impact of polarization on ϵ_*d*_ directly influences N_2_ activation and its subsequent hydrogenation pathways. As shown in Figure 4, for V_2_NPG@In_2_Se_3_, the ϵ_d_ values shift from 0.25 eV (P↑) to 0.09 eV (P↓), aligning with an increase in adsorption strength from −0.51 to −0.69 eV. This downward shift in ϵ_d_ reduces the occupancy of antibonding states, thereby strengthening the V–N_2_ interaction and promoting activation. A similar trend is observed in Co_2_NPG@In_2_Se_3_, where a lower ϵ_d_ in the P↓ state (−1.33 eV) compared to P↑ (−1.19 eV) results in stronger N_2_ adsorption (−0.57 eV vs −0.52 eV). However, Ru_2_NPG@In_2_Se_3_ exhibits different behavior. Despite a downward shift in ϵ_d_ (−1.86 eV for P↓), the adsorption energy weakens (−0.18 eV), suggesting that the d-band center has shifted too low, filling antibonding states and thereby weakening the interaction. This deviation from the expected trend highlights the complexity of the d-band interactions, especially for elements with more delocalized d-states.

Among the studied materials, Ta_2_NPG@In_2_Se_3_ demonstrated the most pronounced polarization-dependent adsorption behavior. In the P↑ state, the relatively high ϵ_d_ of 0.21 eV results in weak N_2_ adsorption (−0.17 eV). However, upon switching to P↓, ϵ_d_ decreases significantly to −0.19 eV, strengthening the adsorption dramatically to −0.99 eV. This substantial shift underscores the critical role of polarization control in tuning the adsorption energetics. Notably, in the pristine state, Ta_2_NPG@In_2_Se_3_ exhibits ϵ_d_ values of 0.19 eV (P↑) and −0.06 eV (P↓), reinforcing its strong polarization-dependent behavior. These results demonstrate that polarization switching effectively modulates ϵ_d_, allowing precise control over the adsorption strength and catalytic performance. The findings further highlight the importance of tuning electronic states through external electric fields to enhance catalytic activity, particularly for systems with strong polarization effects.

To quantitatively capture N_2_ interaction with M_2_NPG@In_2_Se_3_, crystal orbital Hamilton population (COHP) analysis^79^ was performed, and the results are presented in Figure 4. In COHP, negative values indicate bonding interactions, while positive values reflect antibonding contributions. The integrated COHP (ICOHP), calculated by summing contributions from all occupied states up to the Fermi level, provides a net measure of the bonding or antibonding interactions between the metal dimers and N_2_. A more negative ICOHP value reflects a stronger interaction, indicating greater back-donation into the π* orbitals of N≡N. This weakens the N≡N bond, facilitating its activation. Bonding states shifting to higher energies (rightward) suggest weakened bonding due to reduced orbital occupancy, while antibonding states shifting to lower energies (leftward) indicate enhanced metal back-donation, further promoting N_2_ activation.

The differences in ICOHP values between structures reveal the effects of adsorption modes and polarization. For example, V_2_NPG@P↓In_2_Se_3_ adopts a side-on configuration with an ICOHP of −0.59, while V_2_NPG@P↑In_2_Se_3_ favors an end-on configuration with an ICOHP of −0.41. Similarly, Co_2_NPG@P↓In_2_Se_3_ and Co_2_NPG@P↑In_2_Se_3_ exhibit end-on adsorption, showing substantial charge transfer and moderately negative ICOHP values (−0.47 and −0.41), indicating effective d−π* interactions and strong N_2_ activation. In contrast, Ru_2_NPG@P↓In_2_Se_3_ and Ta_2_NPG@P↑In_2_Se_3_ have negligible ICOHP values (−0.02 and −0.01), suggesting weak physisorption of N_2_. However, reversing the polarization significantly increases the ICOHP values (−0.63 and −0.69), signifying full N_2_ activation and making it ready for hydrogenation. Notably, side-on adsorption configurations generally yield more negative ICOHP values and stronger adsorption energies compared to end-on configurations, implying better catalytic performance for NRR in side-on adsorption modes.

To further investigate the differences in N_2_ adsorption, we examined the dipole properties of the V_2_NPG layer. The results indicate that when the V_2_NPG layer is placed on top of P*↓*In_2_Se_3_, an electron transfer of 0.54 e occurs from the V_2_NPG layer to P↓In_2_Se_3_. This transfer partially compensates for the polarization, reducing the built-in electric field within the heterostructure. In this configuration, the potential difference across the heterostructure surfaces is 0.08 eV, with a built-in electric field of 8.31 × 10^7^ V/m (dipole moment = 0.061 eÅ). Conversely, when the V_2_NPG layer is placed on top of P↑In_2_Se_3_, atomic and electronic reconstructions occur, leading to an increased electron transfer of 0.84 e to P↑In_2_Se_3_ to counteract the depolarizing field induced by polarization charges. This significantly raises the potential difference to 2.15 eV and the built-in electric field to 2.07 × 10^9^ V/m (dipole moment = 1.56 eÅ). These reconstructions influence N_2_ adsorption by modifying the binding energies and the availability of adsorption sites. Notably, the strength of the built-in electric field can be estimated by *E* = *P*/ϵ*Sd*, where *P* is the intrinsic dipole moment, ϵ is the dielectric constant, *S* is the surface area, and *d* is the thickness of the heterostructure.

The adsorption of hydrogen (H*), a crucial competing side reaction in the NRR process, not only consumes protons and electrons but also obstructs active sites, as noted in reference.^80^Figure S7 presents the optimized adsorption configurations of H* on M_2_NPG@P↓In_2_Se_3_ and M_2_NPG@P↑In_2_Se_3_ heterostructures, showcasing their interaction with active sites. To evaluate the selectivity of catalysts for the HER and NRR, the free energy difference of H* (Δ*G*^H^*^^) was used as a selectivity descriptor, with the results summarized in Table 1. For V_2_NPG@P↓In_2_Se_3_ and V_2_NPG@P↑In_2_Se_3_, the Δ*G*^H^*^^ were calculated as 0.16 and 0.26 eV, respectively, indicating that HER is less likely to occur on these surfaces compared to other materials, such as Co_2_NPG@P*↑*In_2_Se_3_, Ru_2_NPG@P↑In_2_Se_3_, and Ta_2_NPG@P↓In_2_Se_3_. In the latter materials, the spontaneous HER in one polarization direction increases their susceptibility to H* poisoning on the surface. This strong affinity for H* adsorption reduces their selectivity and limits effective polarization control under reaction conditions, emphasizing the superior selectivity of V_2_NPG@In_2_Se_3_.

### 3.4 Catalytic Activity

To explore the catalytic activity and NRR mechanism, V_2_NPG@P↓In_2_Se_3_ and V_2_NPG@P↑In_2_Se_3_ were selected due to their promising selectivity and controlled hydrogen adsorption behavior. These materials provide an ideal platform for investigating how the polarization direction influences reaction dynamics and how the ferroelectric surface modulates the empty and occupied *d* orbitals, thereby affecting the NRR process.

In the industrial Haber–Bosch process, the ammonia synthesis follows a dissociative mechanism, where the N≡N bond is first cleaved, generating isolated nitrogen atoms that subsequently undergo hydrogenation to form NH_3_. This process requires extreme conditions due to the high energy needed to break the strong triple bond of N_2_. In contrast, electrocatalytic NRR typically proceeds via associative pathways, which can be classified into four main types: distal,^81^ alternating,^82^ enzymatic,^83^ and mixed pathways.^84^ These pathways differ in how nitrogen atoms interact with protons and electrons on the catalyst surface, influencing the selectivity and efficiency.

**Figure 5:**
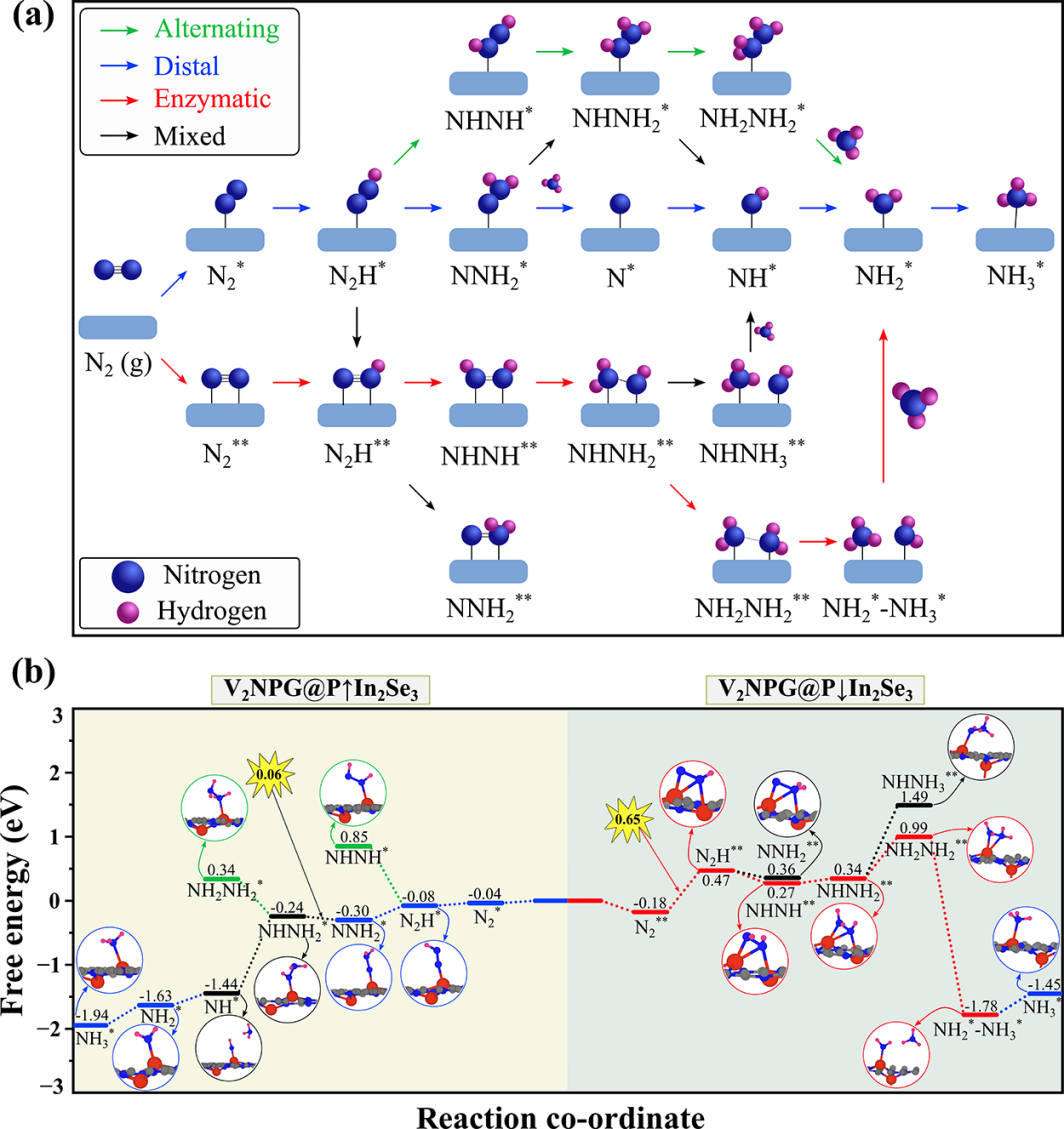
(a) Schematic illustration of potential reaction mechanisms during the NRR process. (b) Gibbs free energy diagrams of the NRR on V_2_NPG@P↑In_2_Se_3_ and V_2_NPG@P↓In_2_Se_3_ heterostructures.

As illustrated in Figure 5a, in the distal pathway, the hydrogenation predominantly occurs at the terminal nitrogen atom, while the surface-bound N atom remains unreacted until the first NH_3_ molecule is released. This stepwise process ensures exclusive NH_3_ production with no byproducts. The alternating pathway, however, involves the simultaneous hydrogenation of both nitrogen atoms, potentially leading to the formation of intermediates such as N_2_H_4_. While industrial N_2_H_4_ production typically relies on the oxidative coupling of NH_3_ in the Haber–Bosch process, direct catalytic approaches for synthesizing N_2_H_4_ from N_2_ have received limited attention,^85,86^ despite significant advancements in NH_3_ synthesis catalysts. The enzymatic pathway follows a distinct side-on binding configuration, where both nitrogen atoms attach to the catalyst surface, allowing for sequential hydrogenation. This orientation offers a unique reaction mechanism, further broadening the possible routes for the electrocatalytic NRR.

Figure 5b (left) presents the free energy profile and optimized geometries for the elementary steps in the distal pathway. Initially, N_2_ adsorbs onto the V_2_NPG@P↑In_2_Se_3_ cluster in an end-on configuration, where it undergoes hydrogenation through proton adsorption and electron transfer, forming the N_2_H* intermediate. In this step, the proton attaches to the distal nitrogen atom, resulting in an elongated N–N bond length of 1.213 Å compared to the preadsorbed N_2_ molecule (1.131 Å). This first step is slightly exergonic with a Δ*G* of −0.04 eV. In the second step, another proton and electron are sequentially added to the N_2_H* species, producing the NNH_2_^*^ intermediate. This step further elongates the N–N bond to 1.314 Å. The reaction is energetically favorable, with a Δ*G* of −0.22 eV. In contrast, the alternating pathway for forming NHNH* from N_2_H* is unfavorable, requiring a high Δ*G* of +0.93 eV, indicating a kinetically challenging step.

In the third step, the NHNH_2_^*^ intermediate is formed via mixed pathways. This step is associated with a small uphill Δ*G* value of 0.06 eV, with further elongation of the N–N bond to 1.412 Å. The alternating pathway involving the formation of NH_2_NH_2_^*^ from NHNH_2_^*^ is also energetically unfavorable under the current conditions. The fourth step involves the release of the first NH_3_ molecule. Successive proton-coupled electron transfers occur at the NH_2_ site of NHNH_2_^*^, leading to a significant downhill Δ*G* of −1.20 eV. Following this, an NH species remains bound to the V atom with a V–NH bond length of 1.664 Å. In the fifth step, the reduction of NH* to NH_2_^*^ occurs with a Δ*G* of −0.19 eV, elongating the V–NH_2_ bond to 1.879 Å. In the final step, NH_2_^*^ reacts with an additional proton and electron to form NH_3_^*^, with a Δ*G* of −0.31 eV, and the V–NH_3_ bond length of 2.155 Å. Overall, the distal pathway is characterized by the protonation of NNH_2_^*^ to form NHNH_2_^*^ as the potential rate-limiting step, given its slightly positive Δ*G* of 0.06 eV among all elementary steps.

After evaluating the catalytic performance of the V_2_NPG@P*↑*In_2_Se_3_ system, we now focus on the opposite polarization direction (V_2_NPG@P↓In_2_Se_3_) to assess its influence on N_2_ activation and hydrogenation. Figure 5b (right) presents the Gibbs free energy profile along with the optimized structures of the elementary steps in the enzymatic pathway, which progresses through six sequential steps. In the first step, the side-on configuration of N_2_ on the V_2_NPG@P↓In_2_Se_3_ cluster undergoes hydrogenation through proton adsorption coupled with electron transfer, forming the N_2_H* species. This step is associated with an uphill Δ*G* of 0.65 eV, with the bond length of N–N in the N_2_H* species being 1.363 Å, compared to the preadsorbed N_2_ molecule (1.173 Å). In the second step, the N_2_H* species is further hydrogenated to form NHNH*, accompanied by a favorable downhill Δ*G* of 0.20 eV. Additionally, NNH_2_ can form with another downhill Δ*G* of 0.11 eV. The third step involves the hydrogenation of NHNH* to , which requires overcoming a small Δ*G* of 0.07 eV. In the fourth step, the NHNH_2_^*^ species undergoes further hydrogenation to form NH_2_NH_2_^*^. This transformation demands an Δ*G* of 0.65 eV, which is higher than the corresponding step in the distal pathway, where Δ*G* is only 0.06 eV. The fifth step sees the release of the first NH_3_ molecule, with the N–N bond lengths increasing progressively throughout the intermediate stages (1.413 Å for NHNH*, 1.430 Å for NHNH_2_^*^, and 1.452 Å for NH_2_NH_2_^*^). This highly favorable step has a downhill Δ*G* of −2.77 eV. The reaction leaves an NH_2_ group bound to the catalyst surface, with a V–NH_2_^*^ bond length of 1.866 Å. Finally, in the sixth step, the  species is hydrogenated to produce the second NH_3_ molecule. This step is endergonic with a Δ*G* of 0.33 eV, and the V–NH_3_ bond length in this step is 2.151 Å.

Furthermore, the kinetics of proton transfer in the potential-limiting steps, specifically  on V_2_NPG@P↑In_2_Se_3_ and  on V_2_NPG@P↓In_2_Se_3_, were analyzed using the Zundel  complex as the solvated proton donor (Figure S11). Initially, the shortest distance between the H atom from  and the N atom at the active site was set to 3.0 Å, which decreased to 2.0 and 1.9 Å in the transition state for V_2_NPG@P↑In_2_Se_3_ and V_2_NPG@P↓In_2_Se_3_, respectively. The calculated activation barriers for these steps are 0.10 and 0.36 eV, suggesting that proton transfer is kinetically feasible under ambient conditions and could be further enhanced at more negative applied voltages.

The N_2_ reduction mechanism on the V_2_NPG@In_2_Se_3_ catalyst, including the reaction barriers for each step and the limiting potentials, is strongly dependent on polarization. While NH_3_ is the primary product in the main pathways, switching the polarization can partially or even completely alter the N_2_ reduction pathway, potentially leading to the formation of different final products. Specifically, switching from the NHNH_2_ pathway on V_2_NPG@P↓In_2_Se_3_ changes the reaction process from an enzymatic to a distal-mixed mechanism. Interestingly, although N_2_H_4_ cannot be directly formed on the V_2_NPG@P↓In_2_Se_3_ catalyst, polarization reversal reactivates the catalytic reduction on the V_2_NPG@P↑In_2_Se_3_ surface. Under these conditions, the N_2_H_4_ molecule can transition from a side-on to an end-on configuration as a byproduct under reasonable limiting potentials. This polarization-dependent switching offers significant potential for fine-tuning the selectivity of N_2_ reduction reactions, enabling the controlled production of both NH_3_ and N_2_H_4_ by modulating the polarization direction of the catalyst.

The NRR selectivity and performance of V_2_NPG@In_2_Se_3_ were evaluated by analyzing the adsorption behavior of key adsorbates (, H*, OH*, and O*) as a function of the applied electrode potential. The equilibrium reaction can be represented as

**Figure 6:**
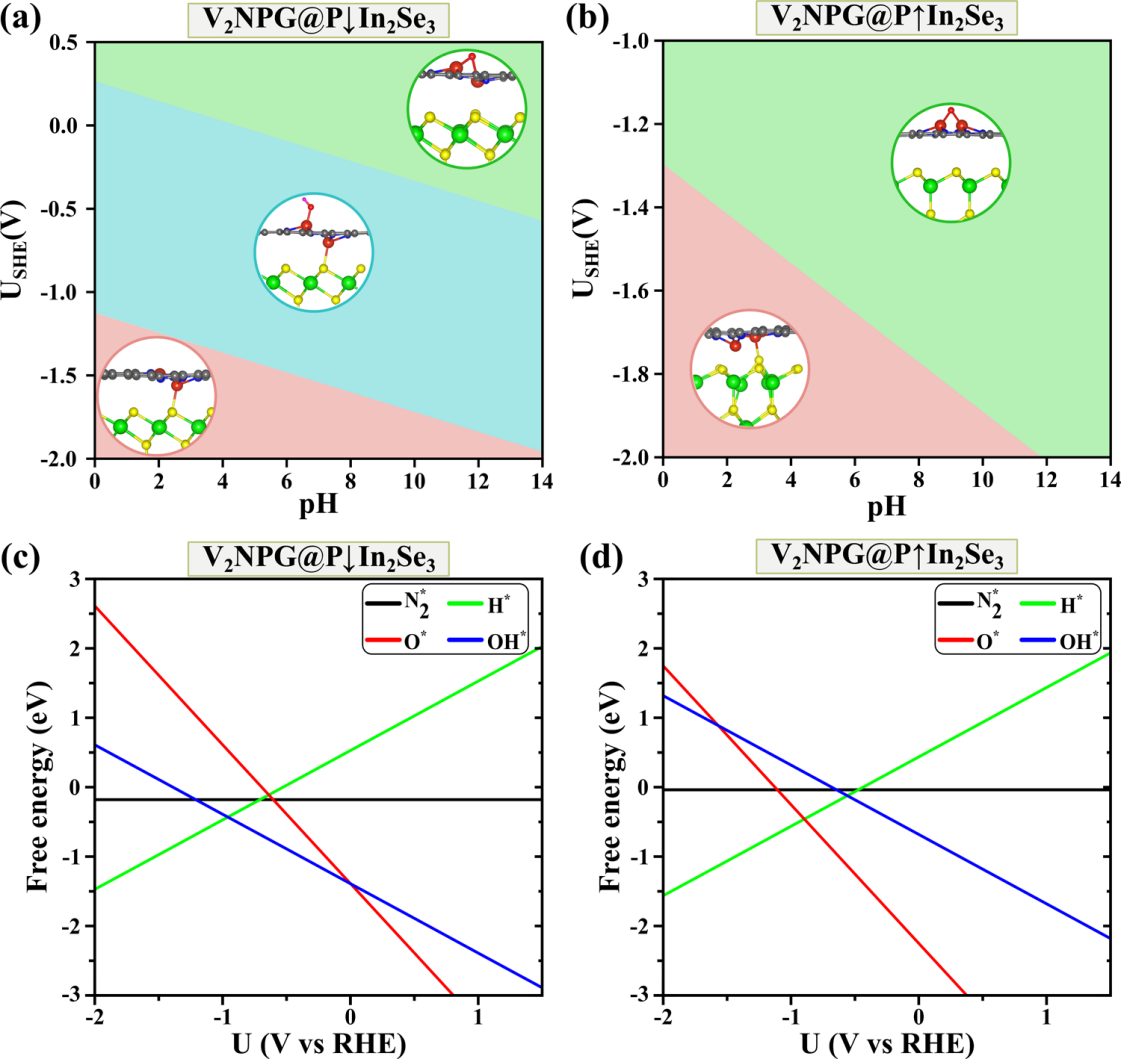
(a) Surface Pourbaix diagrams of V_2_NPG@P↓In_2_Se_3_ and (b) V_2_NPG@P↑In_2_Se_3_. (c) Competitive adsorption of , H*, OH*, and O* on V_2_NPG@P↓In_2_Se_3_ and (d) V_2_NPG@P↑In_2_Se_3_ as a function of the electrode potential.

V_2_NPG@In_2_Se_3_ + *x*H_2_O *⇌* V_2_NPG@In_2_Se_3_@O_x_^*^H_y_^*^ + (2*x* – *y*) + (H^+^ + e^–^) where V_2_NPG@In_2_Se_3_@ denotes the catalyst surface covered by OH*, and O* species.^87^ Notably, a detailed analysis revealed distinct adsorption behaviors for the two polarization states of α-In_2_Se_3_. For the P↓In_2_Se_3_ configuration, the low limiting potential of −0.65 V falls within the potential window where OH adsorption is predominant, while both H and O adsorption are effectively suppressed (Figure 6a,c). This ensures that under operating conditions, the catalyst surface remains largely available for N_2_ adsorption, thereby enhancing the NRR selectivity. In contrast, for P↑In_2_Se_3_, the limiting potential of −0.06 V indicates that O and OH are in competition with N_2_ adsorption, while H adsorption remains suppressed (Figure 6b,d). This suggests that while some oxygen-containing intermediates (OH* and O*) may compete with N_2_ adsorption, the catalyst still retains a significant capacity for selective nitrogen activation. Tuning the applied potential and optimizing the catalytic environment are essential to achieve selective adsorption of  over O* and OH*. Importantly, the results highlight that H adsorption is strongly influenced by the applied potential within the limiting potential range, reinforcing that V_2_NPG@In_2_Se_3_ can effectively mitigate the HER while maintaining an active surface for N_2_ activation.

**Figure 7:**
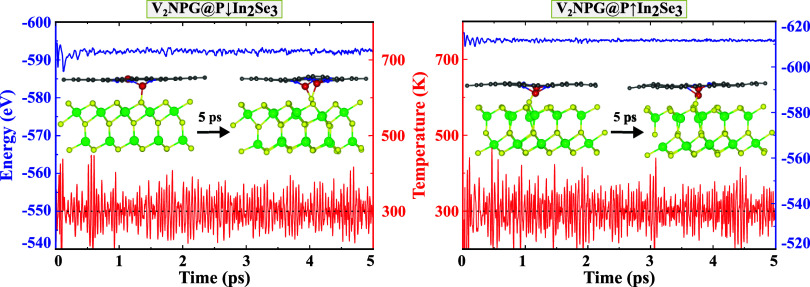
Fluctuations in the total potential energy (blue line) and temperature (red line) during the AIMD simulations of the V_2_NPG@P↑In_2_Se_3_ and V_2_NPG@P↓In_2_Se_3_ heterostructures. The insets display side views of the structures at both the beginning and end of the simulations.

For controllable catalysis to be successful, it is crucial that both the ferroelectric and catalysts themselves maintain good stability. To evaluate the stability of V_2_NPG@In_2_Se_3_, AIMD simulations were conducted at room temperature, as shown in Figure 7. The results indicate that although α-In_2_Se_3_ tends to attract the V atoms of the NPG layer, resulting in a slight reduction in the distance between the two V atoms due to their interaction with α-In_2_Se_3_, the NPG layer preserves its structural integrity, preventing the detachment of the V atoms and their adsorption onto the α-In_2_Se_3_. Atomic fluctuations, resulting from thermal disturbances and the interaction between α-In_2_Se_3_ and the V atoms, are observed in the V_2_NPG@P↓In_2_Se_3_ structure compared to the initial configuration, while the V_2_NPG@P↑In_2_Se_3_ structure remains largely unchanged, retaining its original form. Overall, both structures demonstrate stability over 5 ps, with no metal clustering, phase transitions, or significant energy changes detected.

**Figure 8:**
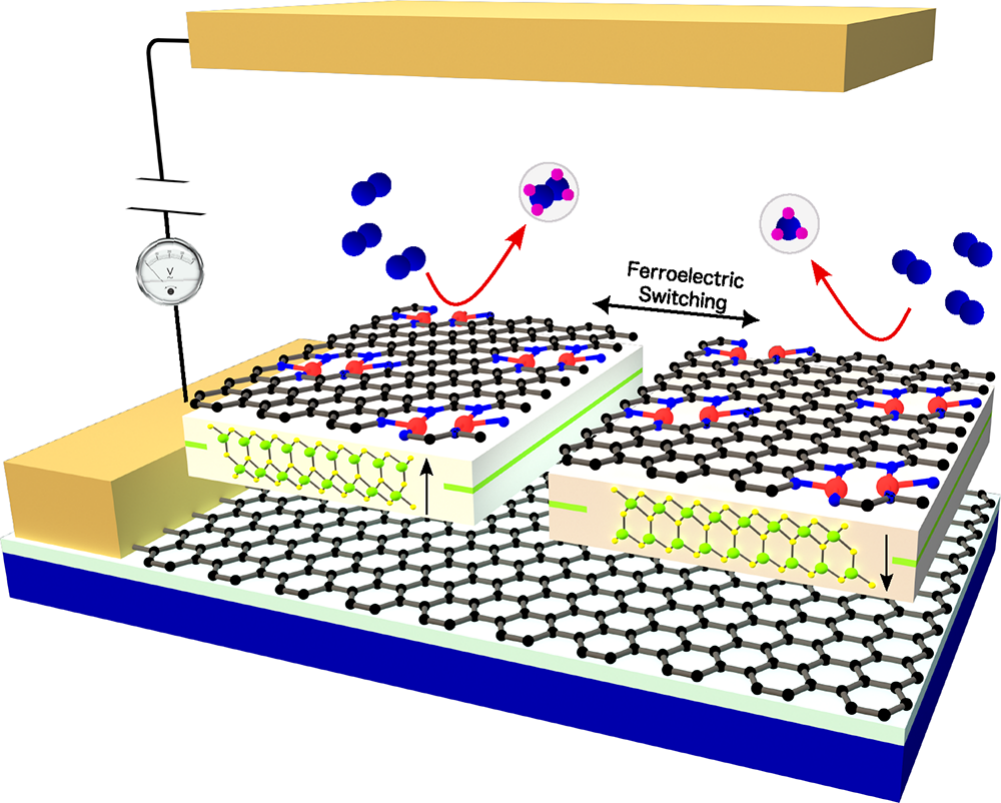
Schematic representation of a ferroelectric-controlled NRR facilitated by the DAC of V_2_NPG@In_2_Se_3_. The heterostructure, comprising V_2_NPG deposited on an α-In_2_Se_3_ monolayer, is placed between two electrodes. By reversal of the bias voltage, the polarization direction of the α-In_2_Se_3_ monolayer can be switched, enabling precise modulation of the catalytic NRR process.

A schematic device design is presented for N_2_ reduction using doped graphene/α-In_2_Se_3_ DACs, as shown in Figure 8. Drawing inspiration from the recently fabricated and predicted ferroelectric diodes based on 2D α-In_2_Se_3_ layers,^28,88^ this design harnesses the unique polarization-dependent catalytic properties of the system. By reversal of the bias direction, ferroelectric switching provides precise control over the reaction pathways and products. Notably, the existence of P↑ and P↓ polarization states in α-In_2_Se_3_ has been experimentally demonstrated, further supporting the feasibility of such devices.^39,71,88^ Furthermore, a comparison of the proposed catalysts with previously reported ones,^22,89−93^ as presented in Table S4, reveals that while V_2_NPG@P↓In_2_Se_3_ exhibits a limiting potential comparable to existing catalysts, V_2_NPG@P↑In_2_Se_3_ achieves a remarkably low limiting potential of −0.06 eV. This exceptionally low limiting potential, combined with the polarization-switchable nature of the proposed catalyst, highlights a unique advantage of this work and underscores its potential significance in the field.

## 4 Conclusions

In conclusion, based on DFT calculations, we investigated the feasibility of polarization-controlled NRR using DACs based on M_2_NPG@In_2_Se_3_ heterostructures. Out of the 27 metals screened, four were identified as highly active for N_2_ activation, with V_2_NPG@In_2_Se_3_ emerging as the most promising candidate. Detailed analysis of its performance revealed a low limiting potential of −0.06 and −0.65 V on P↑In_2_Se_3_ and P↓In_2_Se_3_, respectively, along with tunable reaction pathways and product selectivity enabled by ferroelectric polarization switching. The DAC design enhances the density of active sites and offers controllable catalytic behavior, distinguishing it from traditional SACs. This work highlights the transformative role of ferroelectric materials in catalyst design, paving the way for next-generation, tunable electrocatalysts for NRR and other catalytic reactions. The integration of DACs with switchable ferroelectric materials introduces a paradigm shift toward highly selective and adaptable catalytic systems for diverse applications.

## Supporting Information Available

Information about the PBE + *U*, grand canonical DFT, and the climbing image nudged elastic band (NEB) calculations, the effect of solvation on the free energy diagrams, the electrostatic surface potential (ESP), projected density of states (PDOS) of the α-In_2_Se_3_ monolayer and NPG@In_2_Se_3_ heterostructures, along with all optimized configurations of M_2_NPG@In_2_Se_3_ heterostructures, their structural parameters, cohesive energies, formation energies, dissolution potentials, binding energies, and hydrogen adsorption configurations for both P↑ and P↓ polarization directions of α-In_2_Se_3_ monolayer and comparison of the proposed catalysts from this work with reported ones from the literature (PDF)
