# Supplementary material for: Tunable N2 Fixation Enabled by Ferroelectric Switching in Doped Graphene/In2Se3 Dual-Atom Catalysts
Source: ACS Appl Mater Interfaces. 2025 Feb 27;17(10):15385–97. doi: 10.1021/acsami.4c21092 (PMC11912189; doi:10.1021/acsami.4c21092)
Supplement: Supplementary file 1 — am4c21092_si_001.pdf [file am4c21092_si_001.pdf]

Supporting Information

Tunable N_2 Fixation Enabled by Ferroelectric Switching in Doped Graphene/In_2Se_3 Dual-Atom Catalysts

Mohammad Amin Akhound,[*,†,‡] Maryam Soleimani,[¶] and Mahdi Pourfath[*,†,§]

†*School of Electrical and Computer Engineering, College of Engineering, University of Tehran, Tehran 14395-515, Iran*

‡*CAMD, Department of Physics, Technical University of Denmark, DK - 2800 Kongens Lyngby, Denmark*

¶*Dipartimento di Scienza dei Materiali, Università di Milano – Bicocca, via R. Cozzi 55, 20125, Milano, Italy*

§*Institute for Microelectronics/E360, TU Wien, A-1040 Vienna, Austria*

E-mail: aminakhound@ut.ac.ir,akhound@dtu.dk; pourfath@ut.ac.ir,pourfath@iue.tuwien.ac.at

1 PBE *vs.* PBE+U

The DFT+U approach is widely used to improve the accuracy of DFT calculations for systems with highly localized orbitals. However, the choice of the U value strongly influences the computed energetics, leading to a linear dependence on this parameter and the choice of localized projector functions. For instance, in the $CeO_2 \rightarrow Ce_2O_3$ reduction process, the reaction energy (ΔH) varies from –5.1 eV (U = 0 eV) to –1.9 eV (U = 5.0 eV), while the standard GGA-PBE method gives –4.18 eV, which is in good agreement with experimental measurements (–3.57 to –4.03 eV).[1]

Typically, the U value is selected based on its ability to reproduce the electronic structures (e.g., experimental band gaps) of bulk materials. However, for catalytic applications, where reaction energetics play a critical role, it is often preferable to choose U values that better describe oxidation-reduction energetics.[2,3] In our case, where metal atoms participate in catalytic activity, using bulk-derived U values in a highly dynamic surface environment may not necessarily improve the accuracy of reaction energies.[4,5] Furthermore, GGA-PBE which is used in this study, has demonstrated high accuracy in capturing reaction mechanisms and activity trends observed in experiments.[6]

To assess the impact of the U parameter, we performed benchmark PBE+U calculations on V_2NPG@P$\downarrow$$In_2Se_3$, one of the high-performance catalysts screened out in this work, employing a previously validated U value of 3.5 eV.[7] As shown in Figure S8, the computed theoretical limiting potential as well as the potential-liming step obtained by PBE+U are in good agreement with the PBE results. Considering these factors, along with the computational cost of DFT+U, we have employed standard PBE calculations throughout this study, consistent with previous studies on ferroelectric[8] and dual-atom catalysts.[9]

2 Solvation Effect

To assess the impact of solvation on the free energy diagrams of NRR, we performed additional calculations for $V_2NPG@P{\downarrow}In_2Se_3$ and $V_2NPG@P{\uparrow}In_2Se_3$ using the implicit solvation model implemented in VASPsol.[10] The solvation effects were incorporated by performing single-point energy calculations on the optimized gas-phase structures obtained from standard VASP calculations. The zero-point energy (ZPE) and entropic contributions (TS) were kept consistent with the gas-phase calculations to ensure direct comparability.

As shown in Figure S9, incorporating solvation effects leads to shifts of approximately 0.3 eV in the reaction barriers. However, the potential-limiting steps and the overall trend of catalytic activity remain unchanged on both polarizations, reinforcing the robustness of our conclusions. These results highlight that while solvation effects introduce quantitative corrections, the qualitative activity trends and tunable nitrogen reduction reaction (NRR) insights presented in this study remain valid.

3 Grand Canonical DFT Calculations

Grand canonical density functional theory (GC-DFT) calculations have become a valuable tool for simulating electrochemical processes, especially when studying catalysts under realistic operating conditions. Traditional constant-charge DFT methods keep the number of electrons fixed, which can be limiting when modeling systems at a constant electrode potential. Recent studies have shown that allowing the electron number to vary with the applied potential offers a more realistic description of the electrochemical environment.[11,12] In our study, where nitrogen fixation is tuned by the polarization direction of ferroelectric In_2Se_3, such effects are expected to be significant.

To capture these effects, we employed the recent implementation of GC-DFT in VASPsol,[13] which enables constant-potential calculations by varying the total number of electrons during the self-consistent field (SCF) procedure. In this approach, the electron count on the solute is updated at each SCF step according to

$$N_{\mathrm{e},i+1} = N_{\mathrm{e},i} - C\left(\varepsilon_{\mathrm{F},i} - \mu_{\mathrm{e}}\right), \tag{1}$$

where $\varepsilon_{\mathrm{F},i}$ is the Fermi level of the surface (solute) and μ_{e} is the specified electrochemical potential relative to the bulk electrolyte. The constant C, which approximates the capacitance of the surface, is initially set to 1 eV^{-1} and then updated at each step $i > 1$ according to

$$C = \frac{N_{\mathrm{e},i} - N_{\mathrm{e},i-1}}{\varepsilon_{\mathrm{F},i} - \varepsilon_{\mathrm{F},i-1}}. \tag{2}$$

The grand potential of the system at chemical potential μ_{e} is then defined by

$$\Omega_{\mathrm{tot}} = E_{\mathrm{tot}} - q_{\mathrm{sol}}\,\mu_{\mathrm{e}}, \tag{3}$$

where E_{tot} is the total DFT energy and q_{sol} is the solute charge treated as a variational parameter.

In the GC-DFT calculations, we maintained the same computational parameters as in the constant-charge VASP calculations and incorporated solvation effects to mimic the electrolyte environment. We performed GC-DFT calculations at –0.5 V vs. SHE for all hydrogenation steps of $V_2NPG@P{\downarrow}In_2Se_3$ and compared these results with constant-potential DFT calculations that include solvation effects. As shown in Figure R5, although the limiting potential increased by 0.3 eV, the potential-limiting step remained unchanged, and the overall trends in reaction energetics were very similar. These results confirm that, while GC-DFT can alter reaction barriers, the key conclusion of this study which is the tunability of nitrogen fixation by changing the polarization direction of In_2Se_3 monolayer, remains robust under realistic catalytic conditions.

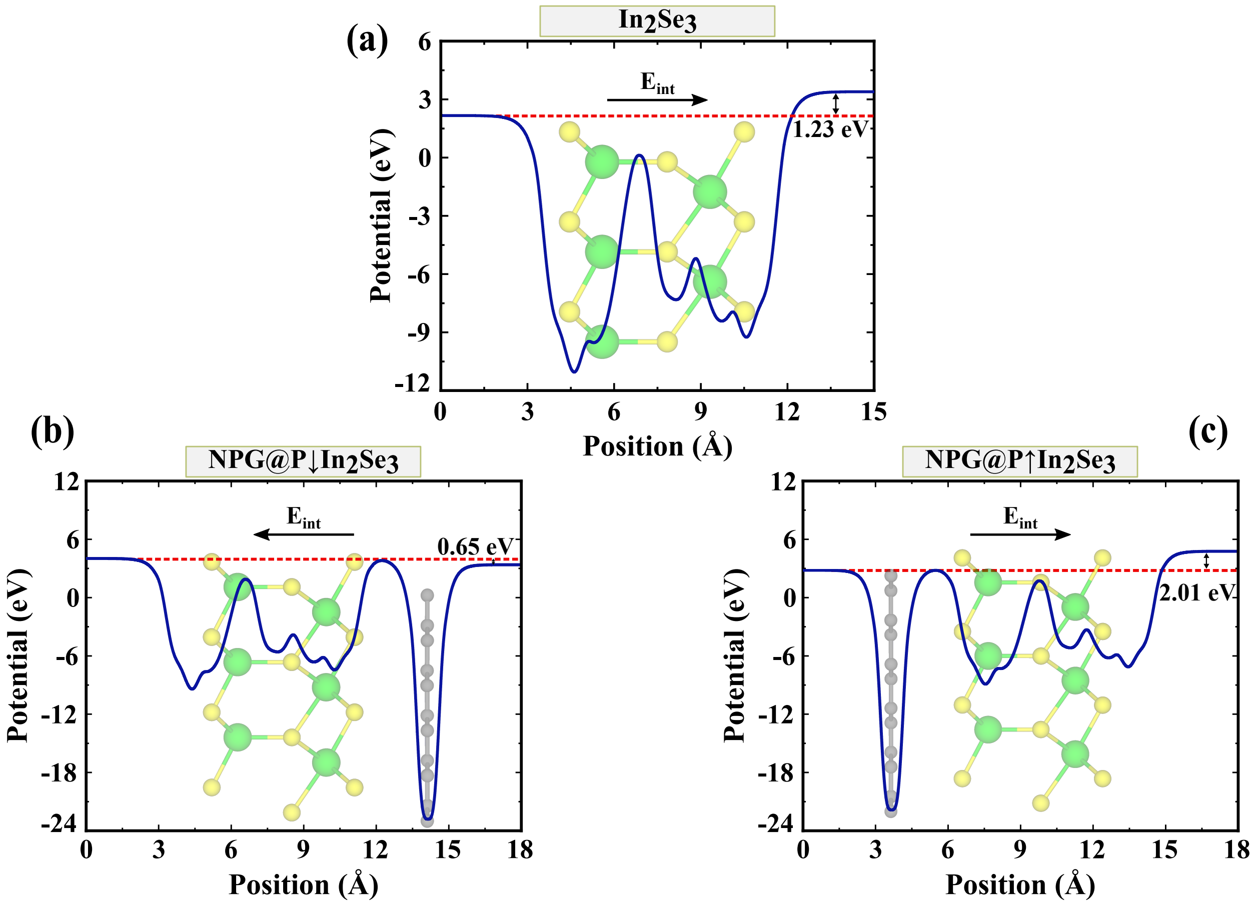

Figure S1: The electrostatic surface potential (ESP) profiles of (a) the In_2Se_3 monolayer, (b) NPG@P↓In_2Se_3, and (c) NPG@P↑In_2Se_3 heterostructures along the direction perpendicular to the surface. Black arrows indicate the direction of the intrinsic electric field in each structure.

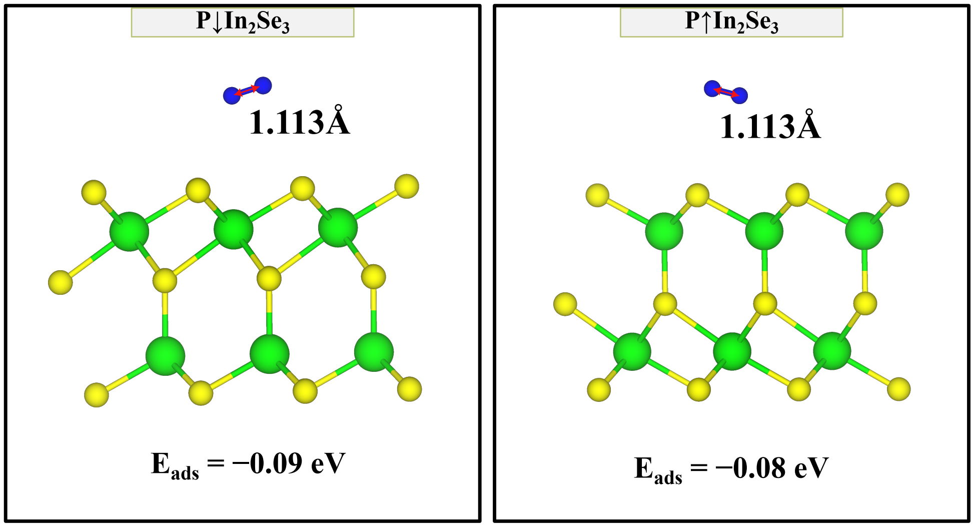

Figure S2: The optimized configurations, adsorption energies, and N–N bond lengths for N_2 adsorption on P↓In_2Se_3 and P↑In_2Se_3 monolayers.

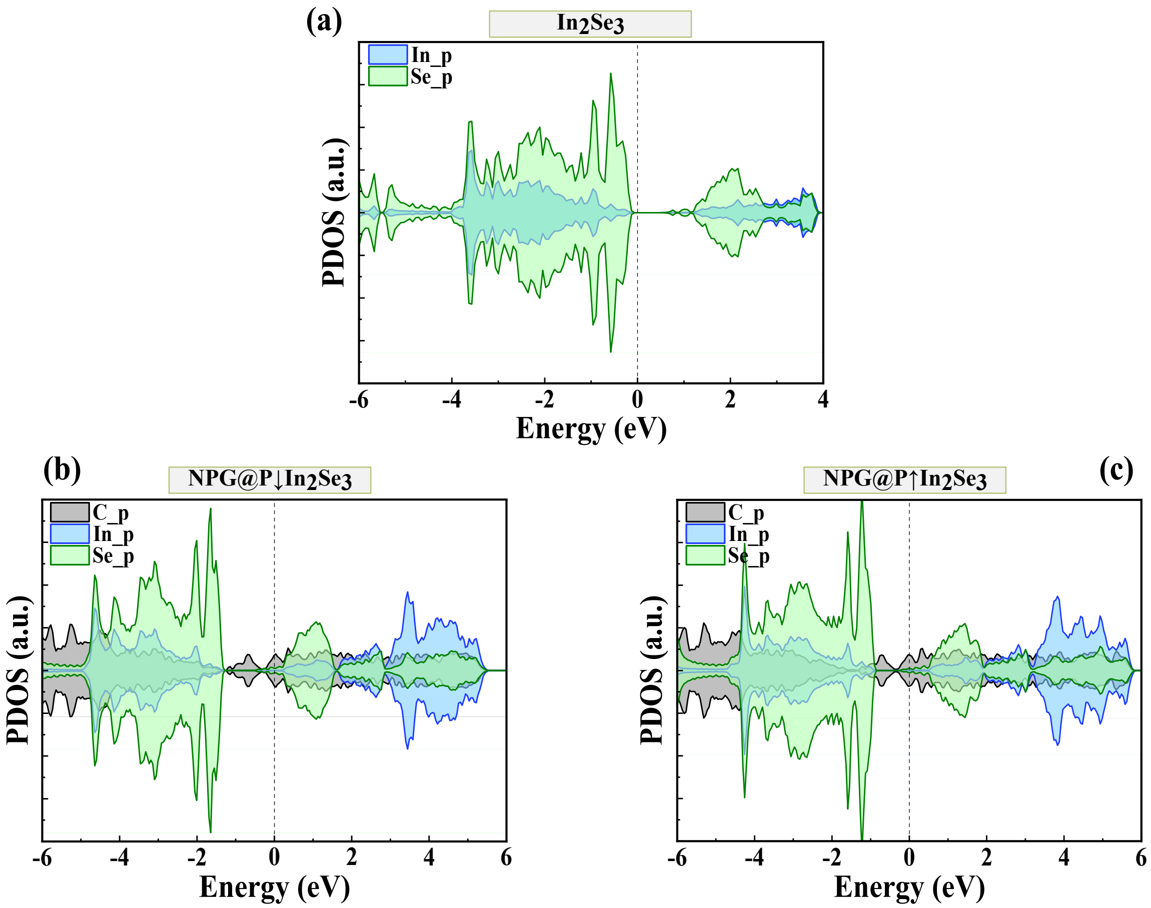

Figure S3: The calculated projected density of states (PDOS) of (a) the In_2Se_3 monolayer, (b) NPG@P↓In_2Se_3, and (c) NPG@P↑In_2Se_3 heterostructures.

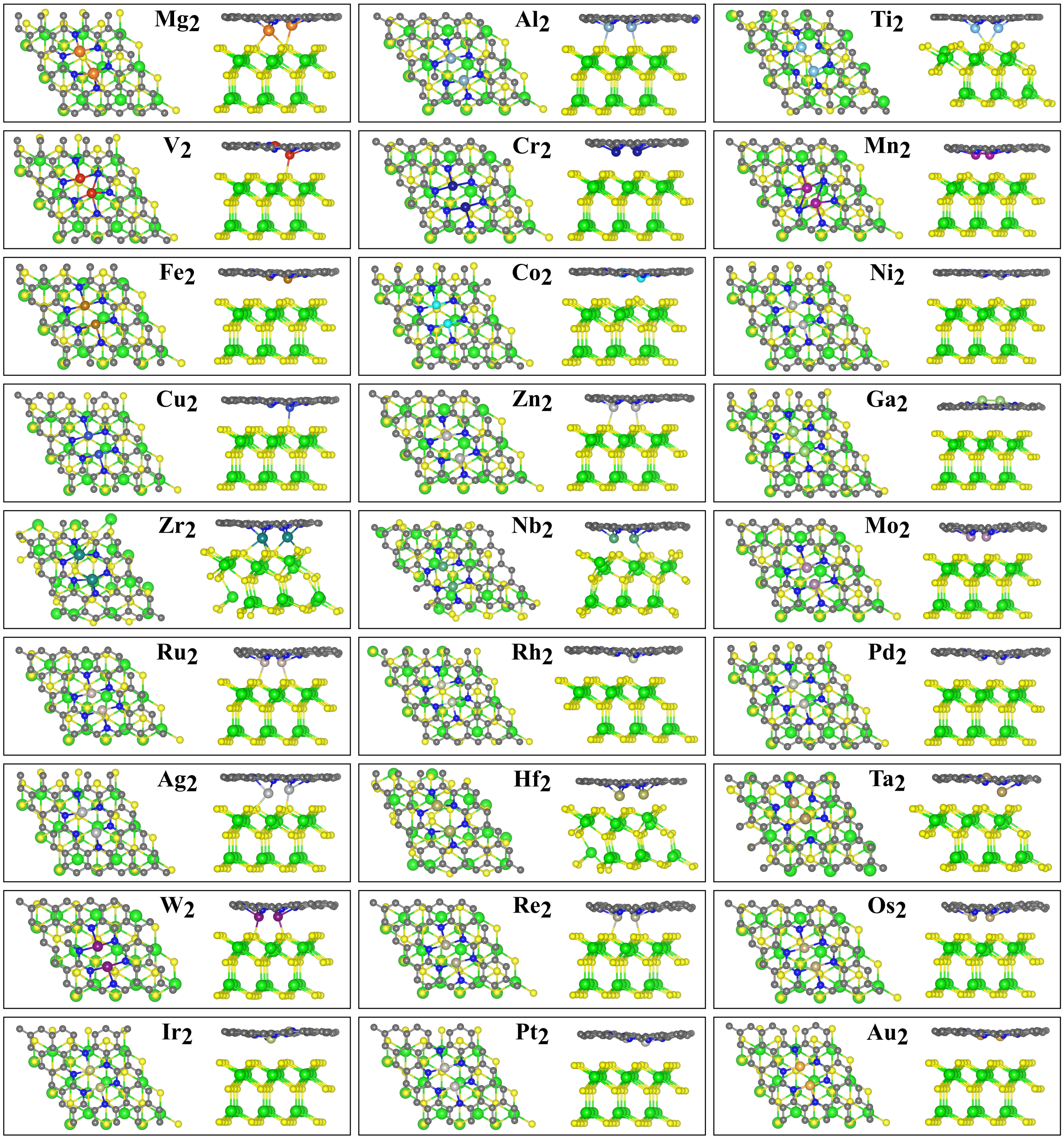

Figure S4: Top and side views of the optimized M_2NPG@P↓In_2Se_3 heterostructures with various metal atom dimers.

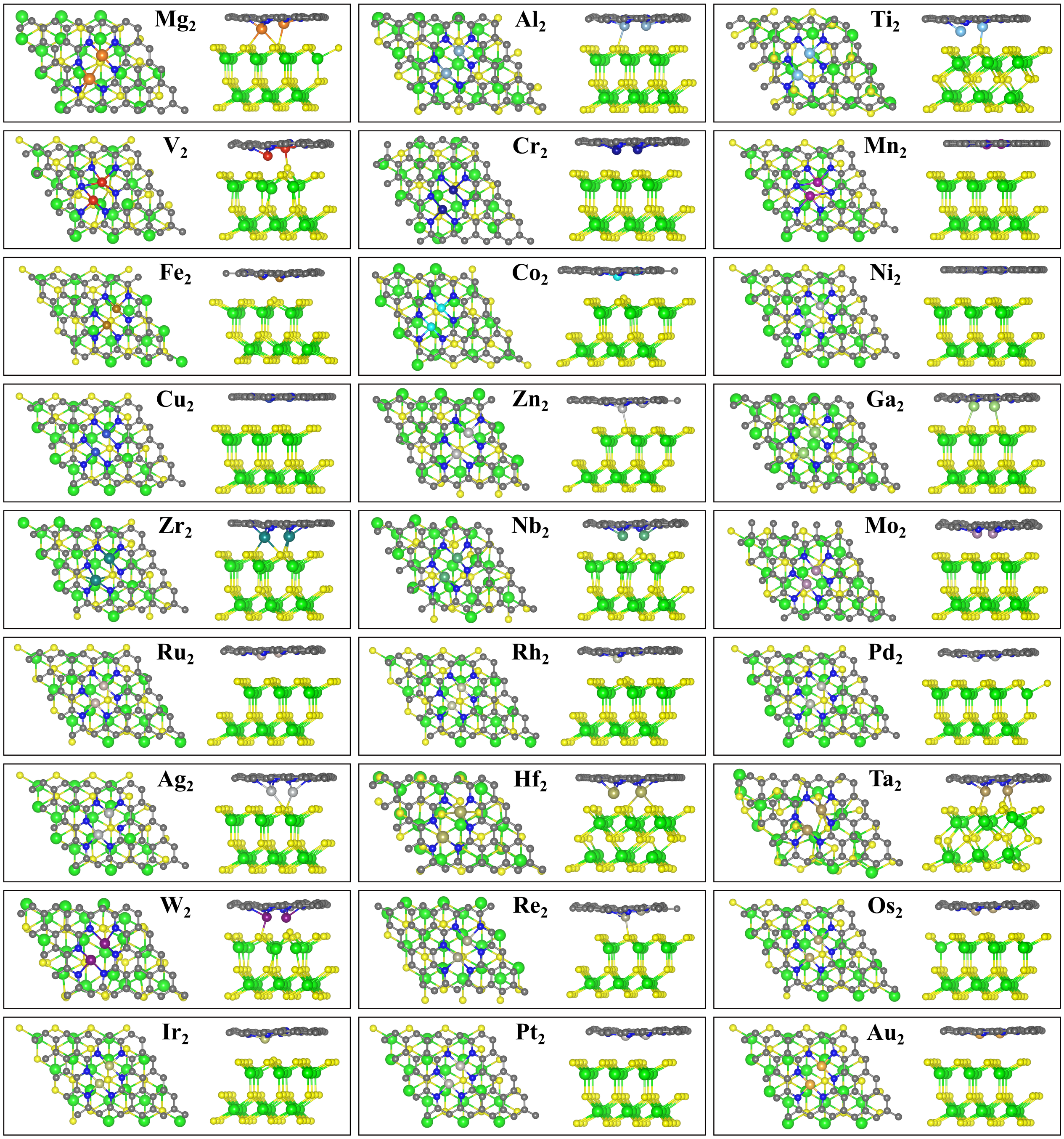

Figure S5: Top and side views of the optimized M_2NPG@P↑In_2Se_3 heterostructures with various metal atom dimers.

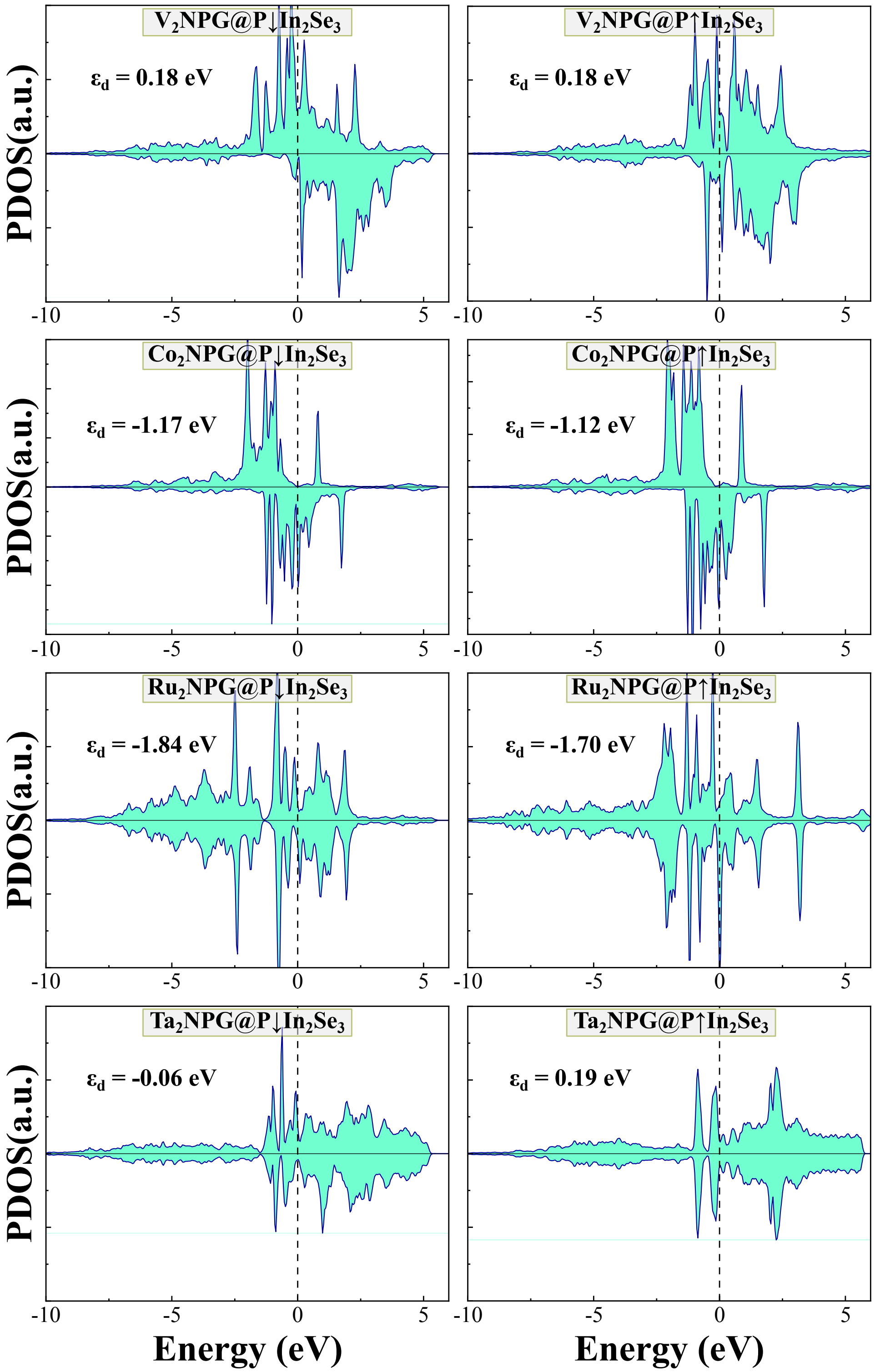

Figure S6: The calculated projected density of states (PDOS) for the d-orbitals of the metal atoms in eight $M_2NPG@In_2Se_3$ (M = V, Co, Ru, Ta) heterostructures with P↑ and P↓ polarization directions of the In_2Se_3 monolayer. The ϵ_d values denote the d-band center of metal atoms relative to the Fermi level, averaged over both spin-up and spin-down states.

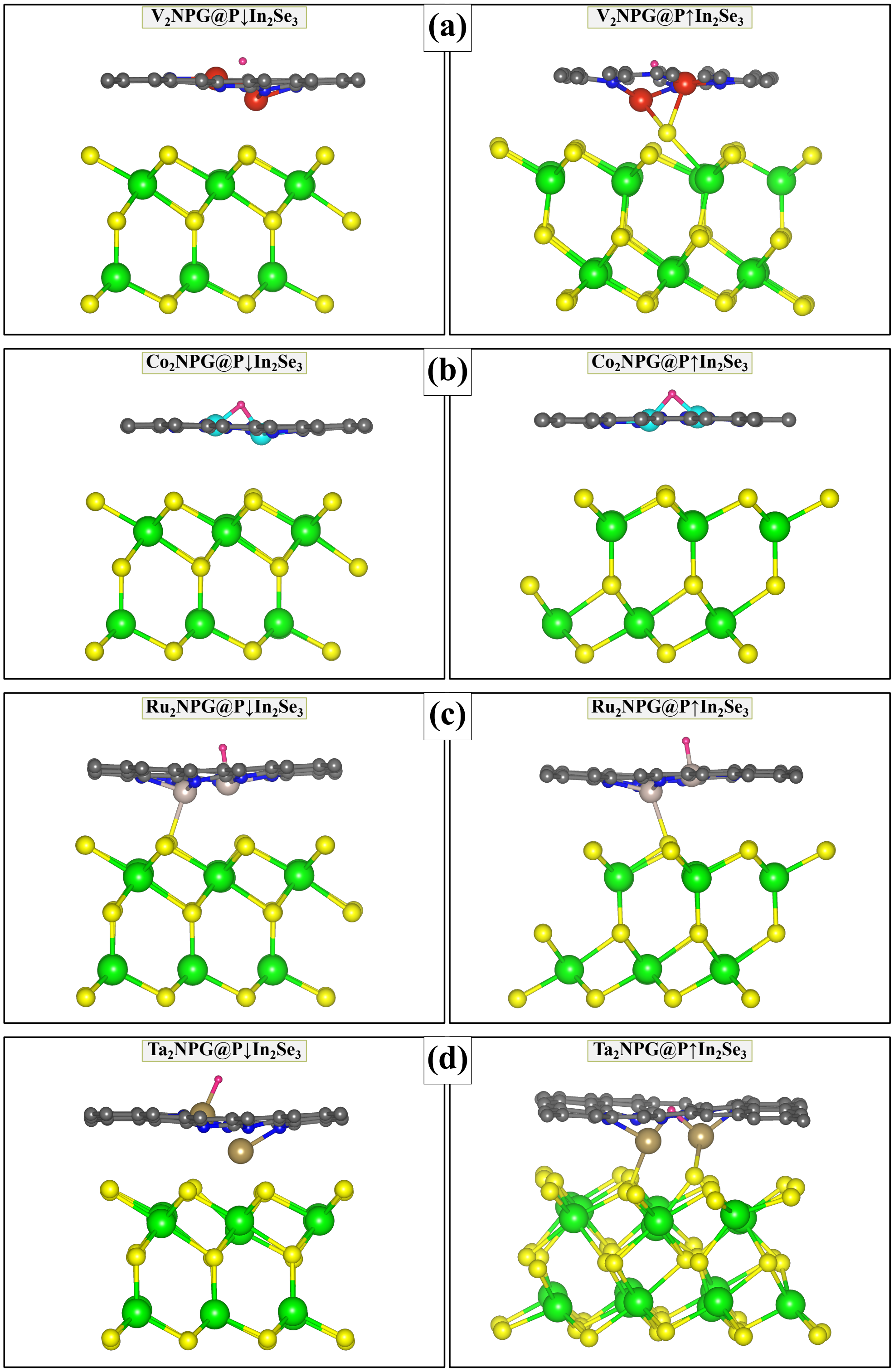

Figure S7: The optimized adsorption configurations of H^* on $M_2NPG@P\downarrow In_2Se_3$ and $M_2NPG@P\uparrow In_2Se_3$ heterostructures with (a) V_2, (b) Co_2, (c) Ru_2, and (d) Ta_2 as active sites.

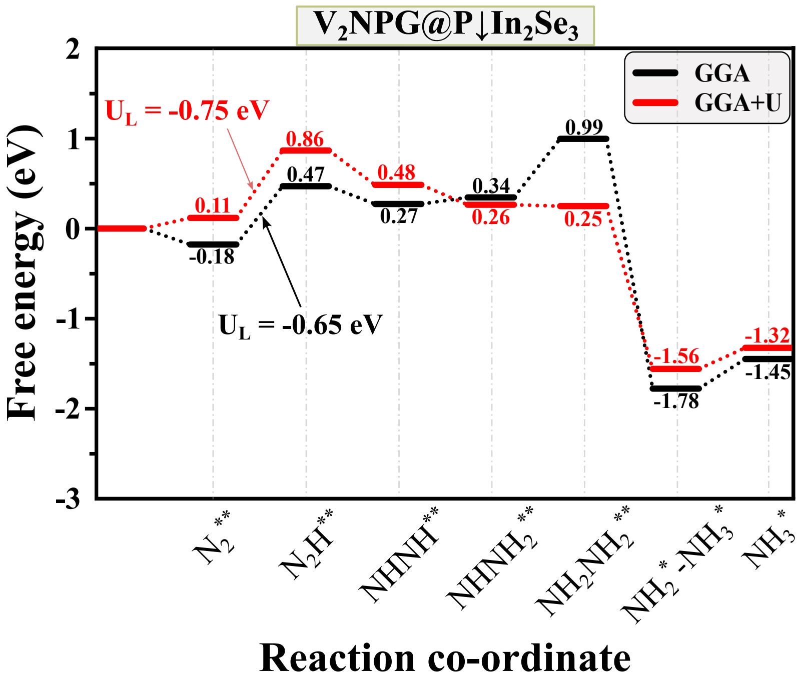

Figure S8: Comparison of the NRR pathways on $V_2NPG@P{\downarrow}In_2Se_3$ by using the PBE and PBE+U methods.

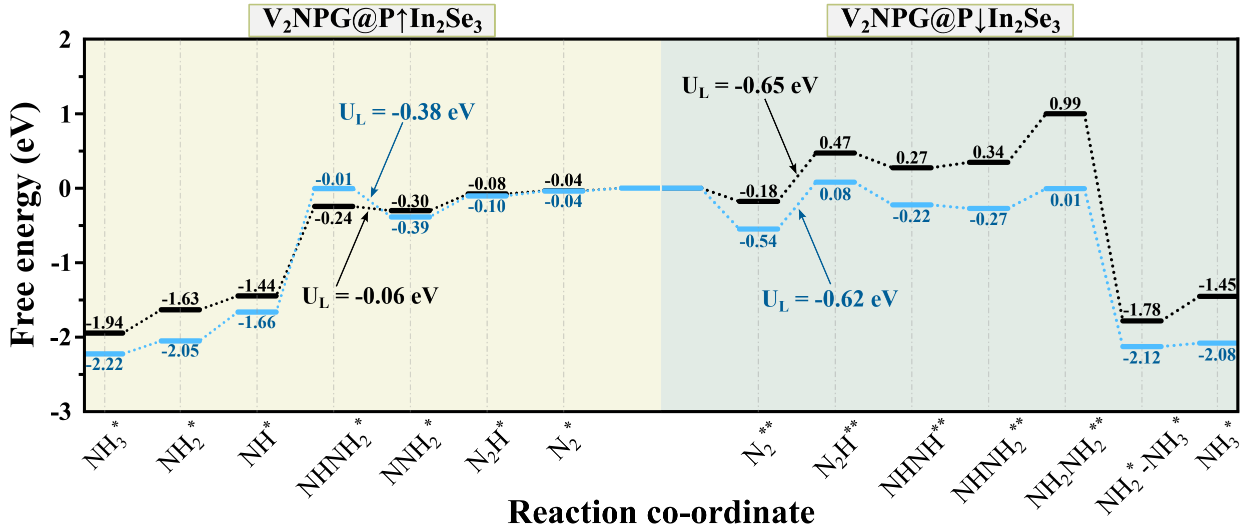

Figure S9: Comparison of the NRR pathways on $V_2NPG@P{\downarrow}In_2Se_3$ and $V_2NPG@P{\uparrow}In_2Se_3$ using solvation-corrected (blue lines) and no solvation accounted (black lines).

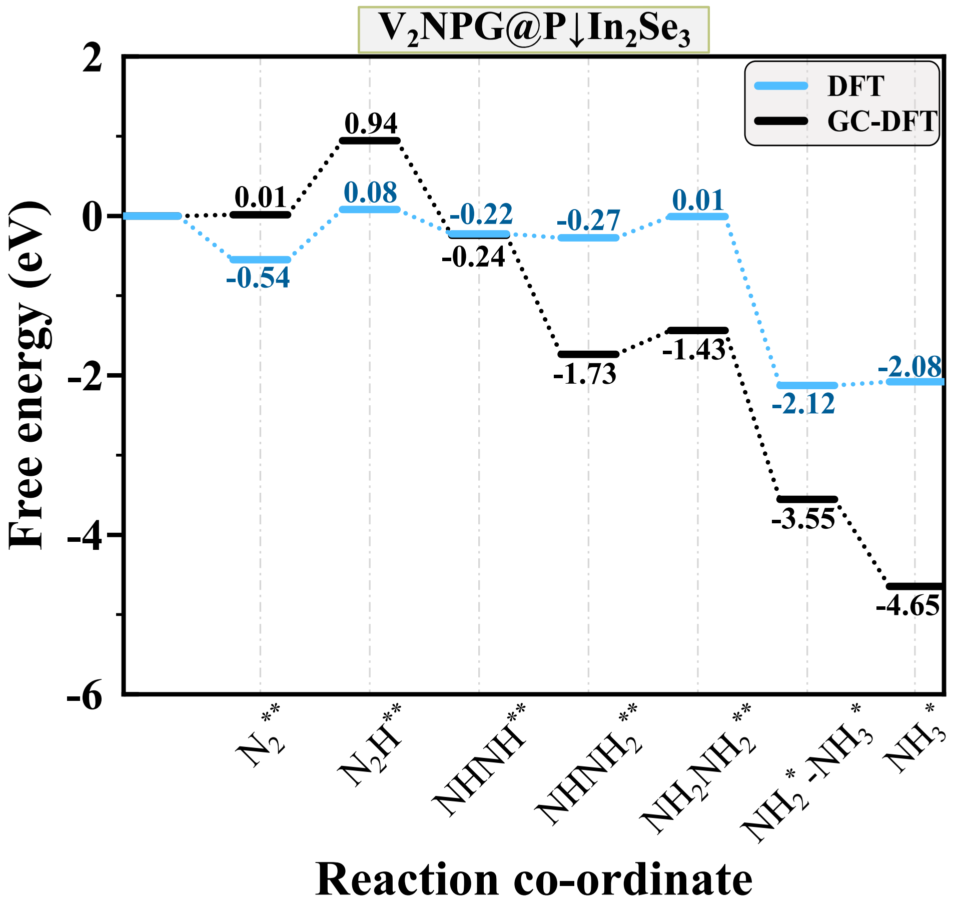

Figure S10: Comparison of the NRR pathways on V_2NPG@P↓In_2Se_3 by GC-DFT at –0.5 V vs. SHE and DFT calculations at constant charge. The solvation effect is considered in both methods.

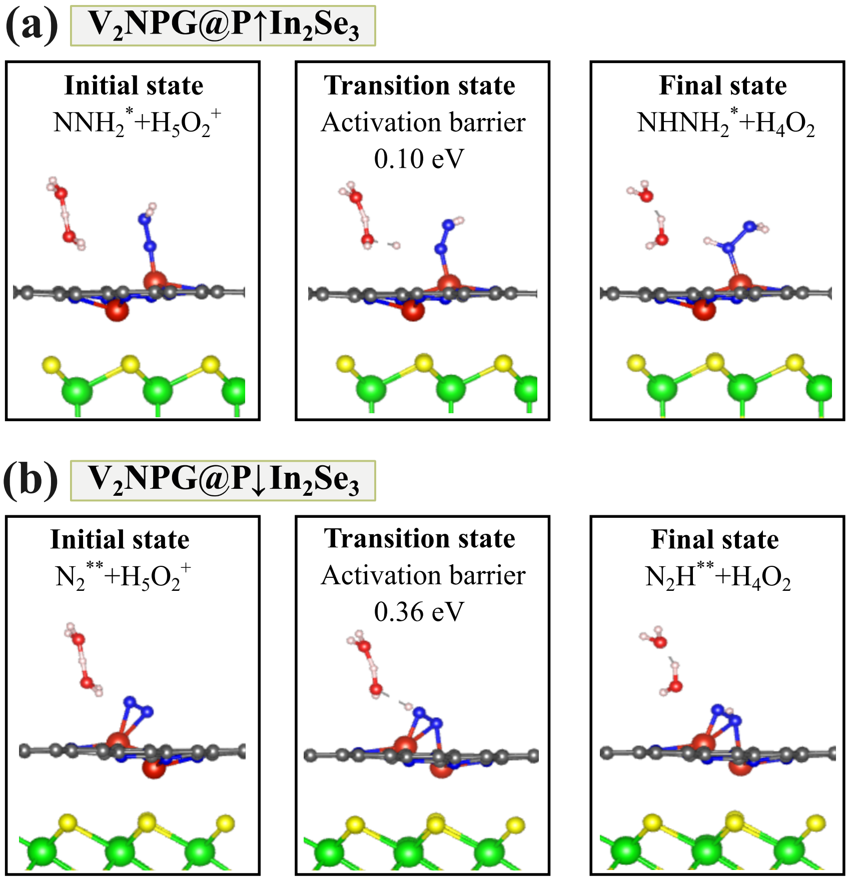

Figure S11: (a) Optimized structures and associated activation barriers for the reduction of NNH_2^* to $NHNH_2^*$ on V_2NPG@P↑In_2Se_3, and (b) N_2^* to $N_2H_6^*$ on V_2NPG@P↓In_2Se_3.

Table S1: Summary of structural parameters for 27 metal atoms in the $M_2NPG@P\downarrow In_2Se_3$ and $M_2NPG@P\uparrow In_2Se_3$ heterostructures. Parameters include the bond length between metal atoms in their bulk phase ($d_{M1\text{-}M2}$), the optimized distance between the metal atoms ($D_{M1\text{-}M2}$), the distance between the first metal atom and the In_2Se_3 monolayer (H_{M1}), the distance between the second metal atom and the In_2Se_3 monolayer (H_{M2}), and the minimum distance between carbon atoms in the NPG layer and the In_2Se_3 monolayer (H_{NPG}).

**Metal**	**$d_{M1\text{-}M2}$ (Å)**	**$D_{M1\text{-}M2}$ @P↓(Å)**	**H_{M1} @P↓(Å)**	**H_{M2} @P↓(Å)**	**H_{NPG} @P↓(Å)**	**$D_{M1\text{-}M2}$ @P↑(Å)**	**H_{M1} @P↑(Å)**	**H_{M2} @P↑(Å)**	**H_{NPG} @P↑(Å)**
Mg	3.16	2.93	1.91	2.52	3.12	2.87	2.06	2.68	3.16
Al	2.85	2.72	2.44	2.44	3.22	2.62	2.59	2.59	3.24
Ti	2.99	2.95	1.88	1.89	2.91	2.71	1.76	2.31	2.93
V	2.59	2.50	3.44	2.39	3.27	2.37	1.31	2.15	2.36
Cr	2.49	2.55	2.38	2.41	3.17	2.48	2.45	2.53	3.19
Mn	2.48	1.85	2.35	2.35	2.98	1.77	3.22	3.29	3.37
Fe	2.46	2.27	2.80	2.49	3.08	2.21	2.53	2.76	2.98
Co	2.48	2.32	2.87	2.54	2.95	2.29	2.72	2.89	3.00
Ni	2.48	2.38	3.05	2.86	3.08	2.37	3.29	3.31	3.36
Cu	2.56	2.45	2.97	2.56	3.13	2.39	3.28	3.31	3.39
Zn	3.01	2.78	2.38	2.39	3.21	2.67	2.50	3.11	3.27
Ga	2.74	2.49	4.21	4.17	3.28	2.50	2.51	2.48	3.23
Zr	3.19	3.15	1.40	1.53	2.90	2.92	1.89	1.98	3.11
Nb	2.88	2.58	1.73	1.71	2.87	2.53	1.70	1.74	2.80
Mo	2.74	1.92	2.38	2.37	3.14	1.85	2.47	2.47	3.16
Ru	2.67	2.21	2.30	2.29	3.15	2.20	2.82	3.09	3.21
Rh	2.72	2.51	3.07	2.35	2.95	2.50	2.37	3.15	3.06
Pd	2.80	2.45	3.00	2.56	3.09	2.41	2.89	2.99	3.23
Ag	2.94	2.74	1.76	2.18	3.35	2.70	1.98	1.88	3.35
Hf	3.13	3.12	1.44	1.61	2.89	3.13	1.65	1.78	3.04
Ta	2.88	2.64	3.26	1.70	2.95	2.64	1.52	1.60	2.62
W	2.75	2.33	2.18	2.20	3.14	2.27	1.69	1.61	2.71
Re	2.75	2.25	2.29	2.26	3.11	2.19	2.42	3.13	3.19
Os	2.70	2.28	2.23	2.21	3.09	2.25	2.82	3.08	3.23
Ir	2.74	2.44	2.70	3.56	3.25	2.47	2.44	3.28	3.07
Pt	2.81	2.45	3.09	2.67	3.04	2.43	2.97	3.08	3.27
Au	2.95	2.44	3.21	3.09	3.26	2.44	3.17	3.23	3.36

Table S2: Computed cohesive energy ($E_c^{calc.}$), formation energy (E_f), dissolution potential (U_{diss}), and binding energy (E_b) of metal atoms in the $M_2NPG@P{\downarrow}In_2Se_3$ and $M_2NPG@P{\uparrow}In_2Se_3$ heterostructures. The total energy of metal atoms in their bulk phase (E_M), number of transferred electrons (N_e) during dissolution, experimental cohesive energy ($E_c^{exp.}$), and standard dissolution potentials (U°_{diss}) are also listed for clarity.

**Metal**	**N_e**	**U°_{diss} (V)**	**E_M (eV)**	**$E_c^{exp.}$ (eV)**	**$E_c^{calc.}$ (eV)**	**E_f@P↓ (eV)**	**E_f@P↑ (eV)**	**U_{diss}@P↓ (V)**	**U_{diss}@P↑ (V)**	**E_b@P↓ (eV)**	**E_b@P↑ (eV)**
Mg	2	-2.37	-1.51	-1.51	-1.51	-4.43	-3.70	-0.16	-0.52	-5.94	-5.21
Al	3	-1.66	-3.74	-3.39	-3.55	-3.88	-3.35	-0.36	-0.54	-7.43	-6.90
Ti	2	-1.63	-7.78	-4.85	-5.51	-5.07	-5.25	0.91	1.00	-10.58	-10.77
V	2	-1.18	-8.94	-5.31	-5.49	-2.66	-3.22	0.15	0.43	-8.16	-8.71
Cr	2	-0.91	-9.47	-4.10	-4.03	-2.63	-2.07	0.40	0.12	-6.66	-6.10
Mn	2	-1.19	-8.89	-2.92	-3.78	-2.25	-1.60	-0.06	-0.39	-6.03	-5.38
Fe	2	-0.45	-7.70	-4.28	-4.42	-2.55	-2.25	0.83	0.67	-6.98	-6.67
Co	2	-0.28	-6.79	-4.39	-4.90	-2.07	-1.81	0.76	0.63	-6.98	-6.72
Ni	2	-0.26	-5.41	-4.44	-4.73	-2.19	-2.06	0.83	0.77	-6.92	-6.79
Cu	2	0.34	-3.72	-3.49	-3.47	-1.89	-1.67	1.29	1.18	-5.36	-5.14
Zn	2	-0.76	-1.10	-1.35	-1.09	-2.88	-2.32	0.68	0.40	-3.97	-3.40
Ga	3	-0.55	-2.91	-2.81	-2.73	-2.19	-2.27	0.18	0.20	-4.92	-5.00
Zr	4	-1.45	-8.52	-6.25	-6.28	-7.58	-5.17	0.44	-0.16	-13.86	-11.45
Nb	3	-1.10	-10.20	-7.57	-6.99	-5.33	-4.85	0.68	0.52	-12.33	-11.85
Mo	3	-0.20	-10.92	-6.82	-6.32	-2.38	-1.85	0.59	0.42	-8.71	-8.17
Ru	2	0.46	-9.26	-6.74	-6.77	-1.46	-0.79	1.19	0.86	-8.23	-7.56
Rh	2	0.60	-7.27	-5.75	-5.71	-1.62	-1.39	1.41	1.29	-7.33	-7.10
Pd	2	0.95	-5.20	-3.89	-3.73	-1.66	-1.50	1.78	1.70	-5.39	-5.22
Ag	1	0.80	-2.70	-2.95	-2.49	-1.26	-0.85	2.06	1.65	-3.75	-3.35
Hf	4	-1.55	-9.96	-6.44	-6.47	-5.77	-6.05	-0.11	-0.04	-12.24	-12.52
Ta	3	-0.60	-11.87	-8.10	-8.19	-2.60	-4.10	0.26	0.77	-10.79	-12.29
W	3	0.10	-13.00	-8.90	-8.46	-1.85	-2.30	0.71	0.87	-10.31	-10.76
Re	3	0.30	-12.42	-8.03	-7.81	-1.61	-0.78	0.84	0.56	-9.42	-8.59
Os	8	0.84	-11.25	-8.17	-8.36	-0.94	-0.15	0.96	0.86	-9.29	-8.51
Ir	3	1.16	-8.87	-6.94	-7.27	-1.07	-1.00	1.52	1.49	-8.34	-8.27
Pt	2	1.18	-6.11	-5.84	-5.50	-1.72	-1.59	2.04	1.98	-7.23	-7.10
Au	3	1.5	-3.21	-3.81	-3.03	-1.06	-0.83	1.85	1.77	-4.09	-3.86

Table S3: Adsorption energies ($\Delta H_{\text{ads}}^{N_2{}^*}$) of N_2 molecule on the stable M_2NPG@P↓In_2Se_3 and M_2NPG@P↑In_2Se_3 heterostructures.

**Metal**	$\Delta H_{\text{ads}}^{N_2{}^*}$**@P↓ (eV)**	$\Delta H_{\text{ads}}^{N_2{}^*}$**@P↑ (eV)**
Ti	-0.15	-0.16
V	-0.69	-0.51
Cr	-0.04	-0.02
Fe	-0.21	-0.14
Co	-0.57	-0.52
Ni	-0.13	-0.09
Cu	-0.15	-0.14
Zn	-0.15	-0.18
Ga	-0.05	-0.07
Nb	-0.09	-0.08
Mo	-0.18	-0.18
Ru	-0.18	-0.61
Rh	-0.12	-0.11
Pd	-0.11	-0.09
Ag	-0.16	-0.13
Ta	-0.99	-0.17
W	-0.18	-0.17
Re	-0.12	-0.23
Os	-0.11	-0.15
Ir	-0.13	-0.12
Pt	-0.15	-0.15
Au	-0.12	-0.11

Table S4: Comparison of the limiting potentials (U_L) of the proposed catalyst from this work with reported values from the literature.

**Catalyst**	**U_L (eV)**	**Reference**
V_2NPG@P↓In_2Se_3	-0.65	This work
V_2NPG@P↑In_2Se_3	-0.06	This work
V_2-Pc	-0.39	[9]
B@InSe	-0.66	[14]
Fe_2@C_2N	-0.76	[15]
Co@g-C_3N_4	-0.61	[16]
Ni_2/g-C_3N_5	-0.27	[17]
W_2@BPN	-0.29	[18]

References

(1) Huang, M.; Fabris, S. CO adsorption and oxidation on ceria surfaces from DFT+ U calculations. *The Journal of Physical Chemistry C* **2008**, *112*, 8643–8648.

(2) Tolba, S. A.; Gameel, K. M.; Ali, B. A.; Almossalami, H. A.; Allam, N. K. The DFT+ U: Approaches, accuracy, and applications. *Density Functional Calculations-Recent Progresses of Theory and Application* **2018**, *1*, 5772.

(3) Hu, Z.; Metiu, H. Choice of U for DFT+ U calculations for titanium oxides. *The Journal of Physical Chemistry C* **2011**, *115*, 5841–5845.

(4) Bhola, K.; Varghese, J. J.; Dapeng, L.; Liu, Y.; Mushrif, S. H. Influence of Hubbard U parameter in simulating adsorption and reactivity on CuO: Combined theoretical and experimental study. *The Journal of Physical Chemistry C* **2017**, *121*, 21343–21353.

(5) Fronzi, M.; Piccinin, S.; Delley, B.; Traversa, E.; Stampfl, C. Water adsorption on the stoichiometric and reduced CeO 2 (111) surface: A first-principles investigation. *Physical Chemistry Chemical Physics* **2009**, *11*, 9188–9199.

(6) Zhang, Z.; Xiao, J.; Chen, X.-J.; Yu, S.; Yu, L.; Si, R.; Wang, Y.; Wang, S.; Meng, X.; Wang, Y.; others Reaction mechanisms of well-defined metal–N4 sites in electrocatalytic CO2 reduction. *Angewandte Chemie International Edition* **2018**, *57*, 16339–16342.

(7) Stahl, B.; Bredow, T. Critical assessment of the DFT+ U approach for the prediction of vanadium dioxide properties. *Journal of Computational Chemistry* **2020**, *41*, 258–265.

(8) Ju, L.; Tan, X.; Mao, X.; Gu, Y.; Smith, S.; Du, A.; Chen, Z.; Chen, C.; Kou, L. Controllable CO2 electrocatalytic reduction via ferroelectric switching on single atom anchored In2Se3 monolayer. *Nature communications* **2021**, *12*, 5128.

(9) Guo, X.; Gu, J.; Lin, S.; Zhang, S.; Chen, Z.; Huang, S. Tackling the Activity and Selectivity Challenges of Electrocatalysts toward the Nitrogen Reduction Reaction via

Atomically Dispersed Biatom Catalysts. *Journal of the American Chemical Society* **2020**, *142*, 5709–5721.

(10) Mathew, K.; Sundararaman, R.; Letchworth-Weaver, K.; Arias, T.; Hennig, R. G. Implicit solvation model for density-functional study of nanocrystal surfaces and reaction pathways. *The Journal of chemical physics* **2014**, *140*.

(11) Tezak, C. R.; Singstock, N. R.; Alherz, A. W.; Vigil-Fowler, D.; Sutton, C. A.; Sundararaman, R.; Musgrave, C. B. Revised Nitrogen Reduction Scaling Relations from Potential-Dependent Modeling of Chemical and Electrochemical Steps. *ACS Catalysis* **2023**, *13*, 12894–12903.

(12) Pedersen, P. D.; Melander, M. M.; Bligaard, T.; Vegge, T.; Honkala, K.; Hansen, H. A. Grand Canonical DFT Investigation of the CO2RR and HER Reaction Mechanisms on MoTe2 Edges. *The Journal of Physical Chemistry C* **2023**, *127*, 18855–18864.

(13) Islam, S.; Khezeli, F.; Ringe, S.; Plaisance, C. An implicit electrolyte model for plane wave density functional theory exhibiting nonlinear response and a nonlocal cavity definition. *The Journal of Chemical Physics* **2023**, *159*.

(14) Ma, B.; Peng, Y.; Ma, D.; Deng, Z.; Lu, Z. Boron-doped InSe monolayer as a promising electrocatalyst for nitrogen reduction into ammonia at ambient conditions. *Applied Surface Science* **2019**, *495*, 143463.

(15) Zhang, Y.; Wang, X.; Liu, T.; Dang, Q.; Zhu, L.; Luo, Y.; Jiang, J.; Tang, S. Charge and spin communication between dual metal single-atom sites on C 2 N sheets: regulating electronic spin moments of Fe atoms for N 2 activation and reduction. *Journal of Materials Chemistry A* **2022**, *10*, 23704–23711.

(16) Liu, X.; Jiao, Y.; Zheng, Y.; Jaroniec, M.; Qiao, S.-Z. Building up a picture of the electrocatalytic nitrogen reduction activity of transition metal single-atom catalysts. *Journal of the American Chemical Society* **2019**, *141*, 9664–9672.

(17) Wu, T.; Fan, X.; Wang, C.; Wu, L.; Bai, Y.; Jia, G. The first principles study of the dual-atom catalyst based on g-C3N5 for efficient nitrogen fixation. *Applied Surface Science* **2025**, *682*, 161648.

(18) Pandiyan, K.; Dhanthala Chittibabu, D. K.; Chen, H.-T. Theoretical Establishment and Screening of Double-Atom Catalysts Supported on Biphenylene for an Efficient Electrocatalytic Nitrogen Reduction Reaction. *ACS Applied Energy Materials* **2024**, *7*, 10758–10769.
